# Thermodynamics and economic feasibility of acetone production from syngas using the thermophilic production host *Moorella thermoacetica*

**DOI:** 10.1186/s13068-017-0827-8

**Published:** 2017-06-12

**Authors:** Stephanie Redl, Sumesh Sukumara, Tom Ploeger, Liang Wu, Torbjørn Ølshøj Jensen, Alex Toftgaard Nielsen, Henk Noorman

**Affiliations:** 1 The Novo Nordisk Foundation Center for Biosustainability, Technical University of Denmark, Kongens Lyngby, Denmark; 2 DSM Biotechnology Center, PO Box 1, 2600 MA Delft, The Netherlands; 3 Department of Biotechnology, Technical University Delft, Delft, The Netherlands

**Keywords:** Syngas fermentation, Syngas, Biomass gasification, Basic oxygen furnace, Natural gas, Techno-economic evaluation, Acetone, Thermophilic fermentation, Biochemical production, Corn stover

## Abstract

**Background:**

Syngas fermentation is a promising option for the production of biocommodities due to its abundance and compatibility with anaerobic fermentation. Using thermophilic production strains in a syngas fermentation process allows recovery of products with low boiling point from the off-gas via condensation.

**Results:**

In this study we analyzed the production of acetone from syngas with the hypothetical production host derived from *Moorella thermoacetica* in a bubble column reactor at 60 °C with respect to thermodynamic and economic feasibility. We determined the cost of syngas production from basic oxygen furnace (BOF) process gas, from natural gas, and from corn stover and identified BOF gas as an economically interesting source for syngas. Taking gas–liquid mass transfer limitations into account, we applied a thermodynamics approach to derive the CO to acetone conversion rate under the process conditions. We estimated variable costs of production of 389 $/t acetone for a representative production scenario from BOF gas with costs for syngas as the main contributor. In comparison, the variable costs of production from natural gas- and corn stover-derived syngas were determined to be higher due to the higher feedstock costs (1724 and 2878 $/t acetone, respectively).

**Conclusion:**

We applied an approach of combining thermodynamic and economic assessment to analyze a hypothetical bioprocess in which the volatile product acetone is produced from syngas with a thermophilic microorganism. Our model allowed us to identify process metrics and quantify the variable production costs for different scenarios. Economical production of bulk chemicals is challenging, making rigorous thermodynamic/economic modeling critical before undertaking an experimental program and as an ongoing guide during the program. We intend this study to give an incentive to apply the demonstrated approach to other bioproduction processes.

**Electronic supplementary material:**

The online version of this article (doi:10.1186/s13068-017-0827-8) contains supplementary material, which is available to authorized users.

## Background

Syngas fermentation for the production of fuels and chemicals has received increasing attention during the last years [1] and is on the way to commercialization [2]. The fermentation of syngas to various biochemicals is based on the use of acetogenic bacteria that can metabolize carbon monoxide, carbon dioxide, and hydrogen [1]. Syngas fermentation processes on their way to commercialization are typically based on carbon monoxide-rich waste gases derived from industry [3]. Another potential source of syngas is reformed natural gas or biogas [4]. The use of gasified, lignin-rich waste biomass would broaden the spectrum of feedstock used for syngas fermentation tremendously, and help replace the fossil carbon. Furthermore, biomass contains a lignin mass fraction of up to 44.5% (for woody biomass) [5]. Lignin is recalcitrant to enzymatic hydrolysis, and its aromatic constituents are not readily consumable by microbes. Alternatively, the lignin fraction could be converted to syngas for biological conversion. However, the production of syngas from biomass would add an additional cost factor to the production process.

A multitude of acetogenic bacteria have been described to date [6]. *Moorella thermoacetica* has been initially used to elucidate the Wood–Ljungdahl pathway (WLP) that enables acetogens to generate energy by fixation of CO_2_ or CO with acetate as main product [7]. Hereby CO can serve as carbon source and electron donor (following Eq. 1), while CO_2_ as carbon source requires another electron donor such as H_2_ (following Eq. 2) [7].

1 $$\begin{aligned}& 4 {\text{CO}} + 2 {\text{H}}_{2} {\text{O }} \to {\text{CH}}_{3} {\text{COOH}} + 2 {\text{CO}}_{2} ;\\ & \quad \Delta_{r} G^{0} = - 196\,{\text{kJ}}/{\text{mol,}} \end{aligned}$$

2 $$\begin{aligned}& 2{\text{CO}}_{2} + 4{\text{H}}_{2} \to {\text{CH}}_{3} {\text{COOH}} + 2{\text{H}}_{2} {\text{O}};\\ & \quad \Delta_{r} G^{0} = - 95\,{\text{kJ}}/{\text{mol}} \end{aligned}$$

The WLP is a well-described pathway [8]. During autotrophic growth, there is no net ATP generated via substrate-level phosphorylation. Energy is solely conserved in chemiosmotic processes [9]. The spectrum of enzymes involved in the electron transport chain as well as the type of cation used to generate the electrochemical gradient differs among acetogens. Although *M. thermoacetica* is relatively well studied, the exact mechanisms of autotrophic energy conservation have not yet been elucidated and different mechanisms have recently been proposed [10–12].

The product range of *M. thermoacetica* is limited to acetate but could be broadened by the introduction of heterologous pathways. Although the development of basic tools enabling genetic engineering has been published [13–15], heterologous expression of industrially relevant product pathways has not been reported for *M. thermoacetica*.

An interesting heterologous product candidate is for example acetone. Since *M. thermoacetica* grows at an elevated temperature (optimum 55–60 °C [7]), products with low boiling point such as acetone (boiling point 56 °C [16]) would allow for easy, inexpensive product recovery through gas stripping. Acetone is used industrially as a solvent and as precursor of plastics and resins [17] and has an annual production of more than 7 million tons with a market growth of 3–4% per year [18]. The acetone market price reached a value below 1 $/kg in 2015 [19]. The US market size for acetone is around 1.4·10^6^ t/year (assuming 90% of capacity) [20]. Acetone could be produced in *M. thermoacetica* by introducing a heterologous acetone pathway, such as the one found in *Clostridium acetobutylicum* [21]. Using an engineered strain of *M. thermoacetica* in which the acetone pathway is expressed, it would be possible to convert syngas into acetone at an elevated fermentation temperature. Heterologous acetone production was recently reported in a mesophilic acetogen [22].

The production of a biochemical such as acetone from a chosen feedstock via a heterologous pathway on a commercial level is dependent on the physiology of the production host, on process technology, and on economics. Biological conversion of syngas to acetone is only thermodynamically feasible if the substrate provides enough energy to cover the energy requirements for cell maintenance and growth [23–25]. Thus, metabolic pathways have to exist to harvest the energy provided by the substrate to generate net ATP. The profitability of the process is dependent on the costs of the substrate and processing costs, as well as the predicted costs to develop the technology.

We have evaluated the process of acetone production by gas fermentation using the thermophilic production host *M. thermoacetica* in a multidisciplinary approach in which we combine the assessment of metabolic and economic feasibility. Techno-economic analysis of gas fermentation in scientific literature is sparse, and to our knowledge, no process analysis has been conducted for syngas fermentation with a thermophilic production strain. Few studies are published for production with mesophilic production strains [26–28].

Furthermore, this study exemplifies the potential of thermophiles for the large-scale production of biocommodities, especially those with a relatively low boiling point such as acetone, ethanol, i-propanol, isoprene, or methyl ethyl ketone [29].

## Methods

In the present study we simulated the production of 30 kt/year acetone (<15% of the annual global growth) from syngas using the acetogen *Moorella thermoacetica* as hypothetical production host. Thermodynamic calculations and calculations regarding bioreactor design were executed with MS Excel.

### Determination of the price for syngas

We have determined the variable cost of production of syngas derived from three different sources, namely industrial waste gases, reformed natural gas, and biomass. We implemented acid gas removal, gas reforming, and reverse water–gas shift reaction (rWGS) to determine, from these diverse sources, the cost of syngas with a composition of comparable CO content. An overview over the process steps and costs are shown in Table 1.

**Table 1:** Overview of syngas production costs

Source of syngas	Process steps to derive cost of production	$/mol CO	$/t CO
BOF gas	Acid gas removal	0.00076	27
Natural gas	Feedstock, steam reforming, rWGS	0.0084	298
Corn stover	Feedstock, logistics, preprocessing, gasification, rWGS	0.015	536

#### Industrial waste gas

The off-gas produced during the basic oxygen furnace (BOF) process of steelmaking is rich in CO, has a low content of contaminants, and is known as a suitable substrate for gas fermentation [30]. For this study, we assumed that basic oxygen furnace gas comes free of charge. According to Handler et al. [31], “steel mill exhaust gases are not currently utilized by any United States mills”. The BOF gas with a CO content of 70 mol% (composition of the gas in Additional file 1: Table S1) undergoes acid gas removal [30] to increase the CO content to 81 mol%. This step leads to a price of 27 $/t CO, which equals 7.6·10^−4^ $/mol CO (Additional file 1: Table S2).

#### Natural gas

Natural gas reforming has been around for several decades. The steam reforming process converts the methane feedstock present in the natural gas to syngas in the presence of steam. Auto thermal reforming (ATR) offers several advantages compared to traditional two-step reforming such as simplicity of design and operation as well as reduced preheating utility consumption [32]. Therefore, based on the values in the literature [32], we assumed that the syngas is generated in a 2:1 (H_2_:CO) ratio utilizing ATR. Subsequently, the syngas exiting the reformer at 1050 °C and 25 bar pressure is cooled prior to being sent to the rWGS reactor, which is one of the most widely explored options [33]. This process was simulated in SuperPro Designer^®^. The process converts CO_2_ and H_2_ to CO under high temperature, based on kinetics obtained from literature [33]. Subsequently, the exiting gases are passed through a condenser at 3 °C to remove the large amount of water generated as a byproduct, while the gases are sent to the fermenter. Additional file 1: Tables S3 and S4 illustrate the breakdown of the operational costs contributing towards the process to achieve the desired conversion. We determined a cost of 298 $/t CO (0.0084 $/mol CO) for the production of syngas from natural gas, of which 146 $/t CO (0.0041 $/mol CO) arise from the cost for natural gas.

#### Biomass-derived waste gas

As a third source of gas we evaluated corn stover-derived syngas. Corn stover would be harvested within a 50 mile radius and transported to the feedstock storage. In our production scenario, 33% of the corn stover in the field is harvested, and the rest has to remain on the land in order to recover the nutrients and prevent excessive erosion [34]. Information regarding feedstock and logistics was obtained from Thompson and Tyner [35]. At the factory, the corn stover bales would be preprocessed (grinding and briquetting). Data related to preprocessing were obtained from Lin et al. [36]. The preprocessed biomass is gasified in a fluidized bed reactor at ca. 870 °C (low temperature gasification). The gasifier unit was selected to have a capacity of 2000 t corn stover briquettes per day. A mass fraction of 52% of the preprocessed feedstock was retained as syngas and as impurities. After removal of impurities, the obtained syngas has a composition of 30% CO, 2% H_2_, 53% CO_2_, and 15% H_2_O (by mass). Equipment details and the syngas composition were acquired from literature [37]. Subsequently, a reverse water–gas shift (rWGS) reaction and a drying step were included to increase the CO content of the syngas. The rWGS reaction and water removal were simulated with SuperPro Designer^®^ [38]. The composition of the rWGS-treated and dried syngas, which is comparable to the composition of the syngas derived from BOF gas and from natural gas is shown in Additional file 1: Table S5. The costs related to syngas production are listed in detail in Additional file 1: Tables S6–S8. The price of corn stover briquettes ready for gasification was determined to be 139 $/t. Gasification was determined to be 10 $/t preprocessed feedstock. Taking gasification, cleaning, rWGS, and drying into consideration, 1 t of preprocessed feedstock is converted to syngas containing 10 kmol CO. The costs of rWGS were determined to be 0.16 $ to produce syngas containing 1 kmol CO. Therefore, the price for syngas was 0.015 $/mol CO (536 $/ton CO).

### Thermodynamics and process reaction

In the process reaction the conversion of carbon and nitrogen sources and other reactants to the products and cell mass is described, where the stoichiometric ratios are determined by the conservation of elements, electrical charge, and energy [24]. The rate of the process reaction (in C-mol/h) is dependent on the specific growth rate and maintenance energy requirements of the microorganism. To obtain the process reaction, firstly the catabolic reaction of product formation was set up, with *ν*_*i*_ as the reaction coefficient of each reactant *i.* Then, the Gibbs energy of the catabolic reaction at standard conditions (*T* = 25 °C, *c*_l_ = 1 M), Δ_*r*_ *G* ^*0*^, was determined using the Gibbs energy of formation, $\Delta_{f} G_{i}^{0}$, of the reactants (Eq. 3).

3 $$\Delta_{r} G^{0} \left[ {{\text{kJ}}/{\text{mol}}} \right] = \mathop \sum \nolimits \nu_{i} \cdot \Delta_{f} G_{i}^{0} .$$

Additionally, the reaction enthalpy Δ_*r*_ *H* ^*0*^ at standard conditions was determined using Eq. 4.

4 $$\Delta_{r} H^{0} \left[ {{\text{kJ}}/{\text{mol}}} \right] = \mathop \sum \nolimits \nu_{i} \cdot \Delta_{f} H_{i}^{0} .$$

The values of Δ_*f*_ *G* ^*0*^ and Δ_*f*_ *H* ^*0*^ are listed in Additional file 1: Table S9. The Gibbs energy of the reaction, Δ_*r*_ *G* ^*0*^, was corrected for the process temperature *T* [K], applying the Gibbs–Helmholtz equation (Eq. 5).

5 $$\begin{aligned}\Delta_{r} G^{T} \left[ {{\text{kJ}}/{\text{mol}}} \right] &= \Delta_{r} G^{0} \cdot \left( {T/298.15\,{\text{K}}} \right) + \Delta_{r} H^{0} \\ \quad& \times (1 - T/298.15\,{\text{K}}), \end{aligned}$$

Δ_*r*_ *G* ^*T*^ was further corrected for the concentration of the gaseous substrate and the concentration of the products in the fermentation broth using Eq. 6 [39], with the concentration of each reactant *i* to the power of its stoichiometric coefficient *ν*_*i*_.

6 $$\Delta_{r} G^{T,c} [{\text{kJ}}/{\text{mol}}] = \Delta_{r} G^{T} + R \cdot T \cdot \ln \left( {c_{i}^{{\nu_{i} }} } \right) \cdot 10^{ - 3} .$$

Subsequently, the Gibbs energy normalized to one mol of carbon source was determined by dividing Δ_*r*_ *G* ^*T,c*^ by the stoichiometric coefficient *ν* of the carbon source.

The energy released by the catabolic reaction is required for cell growth and maintenance. Hence, the anabolic reaction, describing cell mass formation, was set up, using C_1_H_1.8_O_0.5_N_0.2_ (*M* = 24.6 g/C-mol) as an approximation for the ash-free cell mass composition [24].

The energy requirement for autotrophic growth of 1 C-mol cell mass, *a*_*G*_, amounts to approximately 1000 kJ/C-mol [23]. Using this value, the catabolic reaction rate that is required to supply the energy required for the growth of 1 C-mol cell mass, can be derived. The anabolic and catabolic reactions normalized to 1 C-mol of cell mass were combined to obtain the overall reaction of growth, with the stoichiometric coefficients $\nu_{i}^{\text{growth}}$.

To determine the amount of substrate that provides the energy which is required to maintain the cell mass, an approximation for the maintenance energy requirement (*m*_G_) was needed. Tijhuis et al. provided data on the maintenance energy requirement for a large range of aerobic and anaerobic bacteria and concluded that the value of *m*_G_ is mainly influenced by the process temperature and that the influence of carbon source and strain is negligible [40]. An approximation for the temperature dependency of *m*_G_ for anaerobic bacteria according to Tijhuis et al. is shown in Eq. 7.

7 $$m_{\text{G}} [{\text{kJ}/ {\text{C-}}}\text{mol}/{\text{h}}] = 3.3 \cdot {\text{e}}^{{\left[ {\left( { - 69,400/R} \right) \cdot \left( {1/T - 1/298.15\,{\text{K}}} \right)} \right]}} .$$

In this way the catabolic reaction providing enough energy to maintain 1 C-mol cell mass could be formed (with the stoichiometric coefficients $\nu_{i}^{\text{main}}$).

Finally, to obtain the process reaction, the cell mass-specific rates (*q*-rates) of production and consumption of every compound, including the heat released by the reaction, were determined by adding up the catabolic and anabolic sub-reactions (Eqs. 8, 9).

8 $$q_{i} [{\text{C-mol}}/{\text{h}}] = \nu_{i}^{\text{main}} + \mu \cdot \nu_{i}^{\text{growth}} ,$$

9 $$q_{\text{heat}} [{\text{kJ}}/{\text{h}}] = \Delta H_{\text{main}} + \mu \cdot \Delta H_{\text{growth}} .$$

### Bioreactor

A bubble column reactor with a defined height of 30 m and a diameter of 6 m was chosen for the study (reactor volume of 848 m^3^). On the one hand the reactor height should be maximized to reach a high substrate conversion [41], thereby reducing the number of reactors, and thus the capital cost required to meet the desired production metrics. On the other hand, the reactor height was kept well below the practical limit of 40 m of conventional bioreactors [42]. We chose a height to diameter ratio (aspect ratio) of 5, which is a typical value for bubble column reactors in an industrial setting [42].

The pressure in the top part of the reactor (*p*_t_) was set to atmospheric pressure (101,325 Pa). The pressure at the bottom of the reactor (*p*_b_) equals the sum of the pressure in the top part of the reactor (*p*_t_) and the hydrostatic pressure and is therefore a function of the broth volume: using the height of the ungassed liquid column (*h*), the broth density *ρ* (assumed to equal the density of water since the concentration of cell mass and other compounds is relatively low, as will be discussed below), and the gravitational constant *g*, *p*_b_ were determined (Eq. 10). The back pressure asserted by the gas compressed into the reactor was neglected.

10 $$p_{\text{b}} [{\text{Pa}}] = p_{\text{t}} + h \cdot \rho \cdot g.$$

The logarithmic mean pressure (*p*) in the reactor vessel was obtained using Eq. 11 [43].

11 $$p [{\text{Pa}}] = (p_{\text{b}} - p_{\text{t}} )/\ln (p_{\text{b}} /p_{\text{t}} ).$$

#### Gas–liquid mass transfer

The average rate of gas flow (*F*_av_) was obtained using Eq. 12.

12 $$F_{\text{av}} [{\text{m}}^{3} /{\text{h}}] = \left[ {\left( {R_{\text{in}} + R_{\text{out}} } \right) \cdot 0.5 \cdot R \cdot T} \right]/p .$$

The pressure-corrected average superficial gas velocity $v_{\text{gs}}^{\text{c}}$ is dependent on the averaged volumetric gas flow rate through the broth column and the cross-sectional area of the reactor (Eq. 13) [43]. Parameters influencing the average superficial gas velocity $v_{\text{gs}}^{\text{c}}$ (compare Additional file 1: Figure S2) were chosen such that $v_{\text{gs}}^{\text{c}}$ did not exceed 0.15 m/s, which is a conventional value for bubble column reactors with a diameter of up to 10 m [44].

13 $$v_{\text{gs}}^{\text{c}} [{\text{m}}/{\text{s}}] = F_{av} /A/3600.$$

The gas has to be transferred across the gas–liquid interfacial area around the gas bubbles. The liquid-phase mass transfer coefficient *k*_L_ and the interfacial area *a* are both dependent on physical properties and on operation conditions, but are usually merged in their empirical cross-product, *k*_L_ *a* [45]. The value of *k*_L_ *a* was corrected for the process temperature using Eq. 14 and a temperature correction factor *θ* = 1.022 [46].

14 $$k_{\text{L}} a [1/{\text{s}}] = k_{\text{L}} a (20\,^\circ {\text{C}}) \cdot \theta^{{T - 293.15\,{\text{K}}}} .$$

The volumetric mass transfer coefficient *k*_L_ *a* can be derived using Eq. 15 (derivation: see Additional file 1).

15 $$k_{\text{L}} a (20\,^\circ {\text{C}}) [1/{\text{s}}] = 0.32 \cdot \left(D_{i} /D_{{{\text{O}}_{ 2} }} \right)^{0.5} \cdot \left( { v_{\text{gs}}^{\text{c}} } \right)^{0.7} .$$

The diffusion coefficient *D* was obtained by correcting the standard diffusion coefficient *D* ^0^ for the process temperature *T*, using the dynamic viscosity at 298.15 K, *µ* ^0^ (Eq. 16).

16 $$D [{\text{cm}}^{2} /{\text{s}}] = \left( {T/298.15\,{\text{K}}} \right) \cdot \left( {\mu^{0} /\mu_{T} } \right) \cdot D^{0} .$$

The values for *µ* ^0^ at 298.15 K and *µ*_*T*_ at the process temperature were obtained with the Gas Viscosity Calculator online tool [47]. The values for *D* ^0^ were obtained from [48] and are, as well as the values for *µ* ^0^, listed in Additional file 1: Table S10.

The gradient between the concentration of a compound in the gas phase and in the liquid phase serves as the driving force for the gas to overcome the gas–liquid interface [45]. The rate with which the gas enters the liquid phase, the transfer rate (TR), was calculated according to [45], using the dissolved gas concentration at equilibrium (c*) and the average concentration in the liquid phase (*c*_l_) shown in Eq. 17. It was assumed that *c*_l_ of CO equals 1% of c*, due to the constant uptake by the microorganisms.

17 $${\text{TR}} [{\text{mol}}/{\text{m}}^{3} /{\text{h}}] = k_{\text{L}} a \cdot \left( {c^{*} - c_{\text{l}} } \right) = k_{\text{L}} a \cdot 0.99 \cdot c^{*} .$$

The concentration of CO_2_ in the liquid phase was calculated under the assumption that in a steady state the volumetric production rate of CO_2_ by the cell mass, which follows the process reaction and *V*_liq_, equals the transport of CO_2_ from the liquid phase to the gas phase, according to Eq. 18.

18 $$R_{{{\text{CO}}_{ 2} }} [{\text{mol}}/{\text{m}}^{3} /{\text{h}}] = k_{\text{L}} a \cdot (c^{*} - c_{\text{l}} ).$$

The dissolved gas concentration at equilibrium (c*) is dependent on the solubility of the gas (expressed in Henry’s constant). The value of c* was calculated with the mol-fraction of the incoming gas *y*, the temperature-corrected Henry’s law constant *H*_T_ [49], and the logarithmic mean pressure (*p*) using Eq. 19.

19 $$c^{*} [{\text{mol}}/{\text{m}}^{3} ] = H_{\text{T}} \cdot y \cdot p.$$

To obtain the temperature-corrected Henry’s law constant *H*_T_, the constant for solubility in water at standard temperature (*H* ^0^) was corrected for the process temperature *T* using the correction factor *k* (Eq. 20) [49]. The values of *H* ^0^ and *k* are listed in Additional file 1: Table S10.

20 $$H_{\text{T}} [{\text{mol}}/{\text{m}}^{3} /{\text{bar}}] = H^{0} \cdot {\text{e}}^{{[k \cdot (\left( {1/T} \right) - (1/298.15 K))]}} \cdot 10^{3} .$$

The gas holdup of the reactor (*ε*) describes the average volume fraction of the gas in the reactor and was calculated using the superficial gas velocity $v_{\text{gs}}^{\text{c}}$ according to [50] (Eq. 21). We assumed that the headspace volume is negligible.

21 $$\varepsilon = 0.6 \cdot (v_{\text{gs}}^{\text{c}} )^{0.7} .$$

#### Gas compression

The inflow of fresh syngas into the reactor needs to be compressed. The power required to compress the gas was calculated using Eq. 22 (isentropic gas compression) [51]. We assumed an efficiency of 70%, which is the lowest value for isentropic efficiencies [51].

22 $$\begin{aligned} P[W] &= \frac{\gamma }{\gamma - 1} \cdot p_{1} \cdot V_{1} \cdot \left[ {\left( {\frac{{p_{2} }}{{p_{1} }}} \right)^{{\left( {\gamma - 1} \right)/\gamma }} - 1} \right]\\ & \quad \times \left( {100/70} \right).\end{aligned}$$

The syngas stream from the reforming unit enters the compressor with atmospheric pressure (*p*_1_ = 101,325 Pa). The gas is introduced at the bottom of the reactors. Therefore the discharge pressure *p*_2_ equals *p*_b_, the pressure at the bottom of the reactor. The ratio of the specific heat capacity at constant pressure (*c*_p_) and at constant volume (*c*_v_) is designated as *γ* (Eq. 23) [51].

23 $$\gamma = \frac{{c_{\text{p}} }}{{c_{\text{V}} }}.$$

The specific heat capacities at constant pressure (*c*_p_) and at constant volume (*c*_v_) for the gas mixtures were determined using Eqs. 24 and 25 [52].

24 $$c_{\text{p}} = \mathop \sum \nolimits y_{i} c_{{{\text{p}},i}} ,$$

25 $$c_{\text{v}} = \mathop \sum \nolimits y_{i} c_{{{\text{v}},i}} .$$

The values of *c*_*p,i*_ and *c*_*v,i*_ are listed in Additional file 1: Table S11. In order to increase the overall conversion efficiency, a part of the off-gas from the downstream processing unit is recycled to the reactor. Compression of the recycled gas is described in the product recovery section.

#### Product recovery

Off-gases from the fermenter comprise CO_2_, N_2_, H_2_O, acetone, as well as unused CO and H_2_. Process simulators (AspenPlus^®^ [52] and SuperPro Designer^®^ [38]) were used to simulate and validate the costs pertaining to the product recovery and to estimate the energy consumed by various process configurations [38]. The first step in the product recovery scheme was to separate the acetone–water mixture from the gases in the outlet of the fermenter. In order to achieve the desired separation, a condenser was simulated at 283 K and 22 atm (a compressor and cooler precedes the condenser to achieve this condition). Based on the simulated schemes, all CO, N_2_, and H_2_ are removed from the top of the condenser as a gas, while water and acetone are recovered from the bottom as liquid condensate. However, a fraction of CO_2_ is dissolved along with the condensate. The vapor stream from the condenser at 22 atm needs to pass through a turbine, followed by a heater to match the feed conditions to the fermenter. Although the overall scheme is not heat integrated, the heat is recovered from the previous cooling operation (cooler preceding the condenser) rather than introducing fresh utility to supply utility for the heater. Since the three components (acetone, water, and dissolved CO_2_) present in the mixture can be separated by exploiting their relative volatilities, distillation is selected for subsequent purification. In this study, the evaluated schemes were run to achieve higher product concentrations with minimal losses. Simulations were performed on configurations with varying conditions (see Additional file 2). To achieve a higher level of purity, a process scheme with two distillation columns was employed. The first distillation column was used to remove most of the CO_2_ from the liquid mixture, while the second one is utilized to recover the product acetone, with high purity (greater than 99.1%) as a distillate fraction. Additional file 1: Table S12 summarizes the process configurations and the preliminary design choices made to achieve this separation. Additional file 1: Figure S1 shows the process flow diagram of the downstream processing unit.

#### Heat balance

The net rate of heat generation by fermentation was set up (Eq. 26), taking into consideration the heat released by the process reaction, the heat generated during compression of the fresh syngas, and the cooling effect of acetone and water evaporation. The heating/cooling requirements for condensation and distillation from the off-gas were accounted for in the simulation of downstream processing, as described above.

26 $$\Delta H_{\text{net}} = \Delta H_{r} + \Delta H_{\text{gas}}^{\text{comp}} + \Delta H_{\text{acetone}}^{\text{evap}} + \Delta H_{\text{water}}^{\text{evap}} .$$

The contribution of the compression of the fresh gas and the recycled gas to the net heat balance was calculated using Eq. 27 [34].

27 $$\Delta H^{\text{comp}} \,[{\text{kJ}}/{\text{h}}] = R_{i} \cdot c_{\text{v}} \cdot \left( {T - T_{2} } \right).$$

The specific molar heat capacity at constant volume, *c*_v_, was determined using Eq. 25. The temperature of the compressed gas (*T*_2_) was determined with Eq. 28 [51].

28 $$T_{2} = T_{1} \cdot (p_{2} /p_{1} )^{{\left( {\gamma - 1} \right)/\gamma }} .$$

The fresh syngas has a temperature of *T*_1_ = 297 K.

The extent of the cooling effect for compounds entering the vapor phase (Δ*H* ^evap^) was calculated for water and acetone. Δ*H* ^evap^ was determined by multiplying the rate of acetone or water evaporation (in mol/h), respectively, and the heat of vaporization $\Delta H_{i}^{\text{vap}}$ at 60 °C (Eq. 29). For $\Delta H_{i}^{\text{vap}}$ values see Additional file 1: Table S13. The amount of acetone entering the vapor phase per hour equaled the hourly acetone production rate.

29 $$\Delta H^{\text{evap}} \,[{\text{kJ}}/{\text{h}}] = R_{i}^{\text{vap}} \cdot \Delta H_{i}^{\text{vap}} .$$

The rate of water evaporation was determined using Raoult’s law [53]. The value of *p* ^vap^ is listed in Table S13.

30 $$R_{{{\text{H}}_{ 2} {\text{O}}}}^{\text{vap}} = R_{\text{total}}^{\text{out}} \cdot \left( {p_{{{\text{H}}_{ 2} {\text{O}}}}^{\text{vap}} /p_{\text{t}} } \right).$$

After summing up the aforementioned values (Eq. 26), the net heat generated by fermentation, Δ*H*_net_ (in kJ/h), was used to calculate the hourly cooling water requirement *R*_chill_ (Eq. 31) using the molar heat capacity of water *c*_p_ and the temperature difference Δ*T* between the process temperature and the temperature of the chilled water.

31 $$R_{\text{chill}} [{\text{mol}}/{\text{h}}] = \Delta H_{\text{net}} /c_{\text{p}} \cdot \Delta T.$$

#### Product concentration

Since a steady-state system was assumed, the acetone concentration in the fermentation broth was calculated under the assumption that the rate of production (*R*_p_) equals the rate of acetone leaving the reactor with the off-gas (*F*_out_). Equation 32 was used to obtain the partial pressure of acetone (*p*_acetone_) in the off-gas.

32 $$F_{\text{out}} \cdot (p_{\text{acetone}} /p) \cdot \left( {n/V} \right) = F_{\text{out}} \cdot (p_{\text{acetone}} /p) \cdot (p/R \cdot T) = R_{\text{p}} .$$

Using the Henry’s solubility constant of acetone, *H* ^cp^, the acetone concentration in the fermentation broth (*c*_acetone_) could be derived from its partial pressure *p*_acetone_ (Eq. 33).

33 $$c_{\text{acetone}} \, [ {\text{mol}}/{\text{m}}^{3} ] = p_{\text{acetone}} \cdot H_{{T,{\text{acetone}}}} .$$

#### Cell mass concentration and productivity

The amount of cell mass (in C-mol) follows the specific product formation rate (*q*_p_) and the total acetone production rate (*R*_p_) (Eq. 34).

34 $$n_{\text{CM}} [{\text{C-mol}}] = R_{\text{p}} /q_{\text{p}} .$$

Subsequently the cell mass concentration *c*_*CM*_ could be determined (Eq. 35).

35 $$c_{\text{CM}} [{\text{C-mol/m}}^{3} ] = n_{\text{CM}} /V_{\text{liq}} = n_{\text{CM}} /\left( {\left( {1 - \varepsilon } \right) \cdot V_{\text{reactor}} } \right).$$

### Determination of the variable production costs

When translating utilities into costs, the calculations were based on an electricity cost of 0.08 $/kWh, which is the average industrial electricity price in the state of Indiana in 2014 [54]. The cost for chilled water (4 °C) of 0.05 $/m^3^ was derived from the SuperPro Designer^®^ database.

## Results

**Fig. 1:**
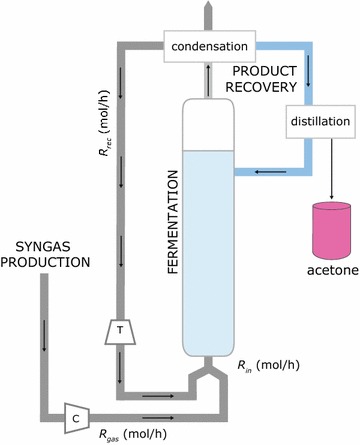
Process overview for the biological production of acetone from syngas. The fresh CO-rich gas is mixed with recycled gas and introduced into the reactor at the flow rate *R*_in_. The recycled gas leaves the condensation unit with high pressure and is passed through a turbine (T) to adjust the pressure and to generate electricity, while syngas requires compression (C). The bubble column reactor has a height of 30 m and a diameter of 6 m. CO entering the liquid phase is assumed to be completely converted to acetone by the production strain *Moorella thermoacetica*. Acetone leaves the reactor with the off-gas; acetone and evaporated water are condensed and then separated in a distillation step. The water from the product recovery is recycled in the reactor

**Fig. 2:**
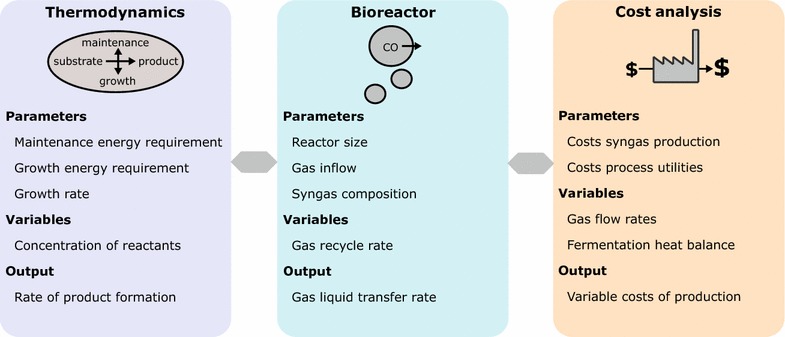
Study approach. The presented model to estimate the variable costs of acetone production from CO with *M. thermoacetica* can be broken down into 3 parts. Thermodynamics: assuming an energy requirement of 62 kJ/C-mol/h for maintenance, and 1000 kJ/C-mol for growth, and a specific growth rate of 0.10 h^−1^, the process reaction was established. The process reaction, which describes the rate of conversion of CO, H_2_O, and the nitrogen source to CO_2_, cell mass, and acetone, is depending on the concentration of the reactants. The concentration of the gases and acetone in liquid was determined by taking gas–liquid mass transfer limitations into account. Bioreactor: the reactor dimensions (30 m height, 6 m diameter), the gas inflow rate *R*_in_, and the composition of the syngas were fixed. The gas transfer rate into the liquid under the chosen process conditions was determined depending on the ratio of fresh and recycled gas. The gas transfer rate determines the amount of substrate that is available to the cell mass and was used as input in the process reaction. For the thermodynamic calculations and calculations on gas–liquid mass transfer, the process temperature of 60 °C was taken into account. Cost analysis: the production rate of the whole plant was set to 30 kt/year and determined eventually the sizing of the plant as well as the variable costs of production

This study has been based on a hypothetical facility located in the Midwest of the US. The syngas is fed into a bubble column reactor with a height of 30 m and diameter of 6 m in which the production strain converts the gaseous substrate into acetone as the sole product. Acetone leaves the reactor with the off-gas and is recovered in subsequent condensation and distillation steps (Fig. 1). The annual production was set to 30 kt/year, with 330 days per year plant operation, the production has to be at least 3.79∙10^3^ kg/h in order to reach the desired production metrics. The study was conducted in a multi-level approach consisting of the following three parts: bacterial physiology, bioreactor design, and cost analysis (Fig. 2). Those parts were implemented such that the output of thermodynamic calculations is directly connected with the reactor design and cost estimations and vice versa.

Prior to the model implementation we studied the metabolic pathways of *M. thermoacetica* to analyze which components of the syngas can serve as substrate for acetone production and the theoretical conversion yield as described in more detail below. We determined the cost for three different syngas sources and rejected those that are, based on the feedstock unit cost and the theoretical yield, not economically viable.

In the first part of the model (Fig. 2), we applied the principle of anaerobic product formation, maintenance, and growth to derive the substrate conversion rate. In the second part, the bioreactor design was taken into account to determine the amount of substrate that is available to the cell mass. We identified parameters related to fermentation and plant sizing and estimated in the third part of the study the variable costs of production.

### ATP yield for production of acetone

**Fig. 3:**
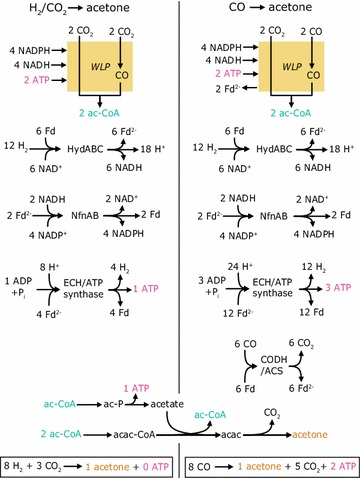
ATP generation for acetone production as the sole end product. According to the mechanism of energy conservation for autotrophic growth in *M. thermoacetica*, 1 mol ATP, 2 mol NADH, and NADPH each, are required in the Wood–Ljungdahl pathway (WLP) for the fixation and conversion of CO_2_ to acetyl-CoA. When CO_2_ serves as carbon source, reduced ferredoxin is required to reduce CO_2_ to CO. This mol reduced ferredoxin which is additionally available to the cell when CO serves as electron donor and carbon source, which explains the ATP generation when CO serves as substrate. *acac* acetoacetate, *acac-CoA* acetoacetyl-CoA, *ac-CoA* acetyl-CoA, *ac-P* acetyl phosphate, *ATP* adenosine triphosphate, *CODH/ACS* CO dehydrogenase/acetyl-CoA synthase, *ECH* membrane-associated [NiFe]-hydrogenase, *Fd* ferredoxin (oxidized form), *Fd* ^*2−*^ ferredoxin (reduced form), *HydABC* electron-bifurcating ferredoxin- and NAD-dependent [FeFe]-Hydrogenase, *NAD* ^*+*^ nicotinamide adenine dinucleotide (oxidized form), *NADH* nicotinamide adenine dinucleotide (reduced form), *NADP* ^*+*^ nicotinamide adenine dinucleotide phosphate (oxidized form), *NADPH* nicotinamide adenine dinucleotide phosphate (reduced form), *NfnAB* electron-bifurcating transhydrogenase

CO, CO_2_, and H_2_ are the main components of syngas. *M. thermoacetica* can grow autotrophically with CO as carbon source and electron donor, or with CO_2_ as carbon source and H_2_ as electron donor [7]. Whether CO and H_2_/CO_2_ can serve as substrate for the production of acetone is dependent on the net ATP production of the conversion. Figure 3 shows an overview of the pathways from H_2_/CO_2_ or CO, respectively, to acetyl-CoA and acetone. Based on the mechanism of energy generation in *M. thermoacetica* proposed by Schuchmann and Müller [11], no ATP would be produced per mol acetone for growth on H_2_ and CO_2_. However, for growth on CO as carbon and energy source, 1 mol ATP would be gained per mol of acetone. Hence, as there is no net gain of ATP when CO_2_ serves as carbon source with H_2_ as electron source, we assumed that only CO can serve as substrate for the production of acetone. Alternative scenarios, which would allow the utilization of H_2_/CO_2_ alongside CO, are addressed in the discussion section. When CO serves as the only carbon source, 1 mol acetone is produced from 8 mol CO; the theoretical carbon yield is therefore 0.125 mol acetone/mol CO.

### Syngas sources

The CO-rich gas feed can be derived from various sources. We estimated the cost of syngas with a CO content of 33–38 mol/m^3^ derived from industrial waste gas, natural gas, and biomass. The theoretical conversion yield was used to identify syngas sources that have the potential to be utilized for an economically viable biological production of acetone.

As shown in Table 1, we estimated a cost of 7.6·10^−4^ $/mol CO (27 $/t CO) for off-gas from a BOF process in the steelmaking industry after acid gas removal. With a carbon yield of 0.125 mol acetone/mol CO, the costs for the substrate would equal 0.11 $/kg acetone, which is 10–25% of the recent acetone selling price. For syngas derived from natural gas (0.0084 $/mol CO or 298 $/t), a conversion of CO to acetone by the maximum theoretical yield of 0.125 mol acetone/mol CO would lead to a substrate cost of 1.16 $/kg acetone, which is above the recent acetone selling price. For the production of syngas with a high CO content from corn stover, multiple process steps are required. The price for syngas was determined to be 0.015 $/mol CO (536 $/ton CO). Taking into account the theoretical conversion yield, the substrate-related cost of 2.1 $/kg acetone would make the process economically uninteresting. Therefore, only syngas derived from BOF gas has the potential to be economically viable.

### Thermodynamics and bacterial physiology

For the simulated scenario, the process temperature was set to 60 °C, which is within the optimal range for *M. thermoacetica* (55–60 °C) [7]. Growth profiles of *M. thermoacetica* on CO have previously been published by Kerby and Zeikus [55]: We extracted data from Fig. 2 of the publication (using WebPlotDigitizer [56]), and determined a specific growth rate of around 0.10 h^−1^, which we used for this study.

The Gibbs free energy released by the reaction of CO to acetone amounts to −323 kJ/mol under standard conditions (Eq. 36). The standard molar Gibbs energy of formation Δ_*f*_ *G* ^0^ of the single reactants is listed in Additional file 1: Table S9.

36 $$8 {\text{CO}} + 3 {\text{H}}_{ 2} {\text{O}} \to 1 {\text{C}}_{ 3} {\text{H}}_{ 6} {\text{O}} + 5 {\text{CO}}_{ 2} ;\quad \Delta_{r} G^{0} = - 3.22.8\,{\text{kJ}}/{\text{mol}} .$$

The Gibbs energy of the reaction was corrected for the process temperature of 60 °C (using Eq. 5) to obtain Δ_*r*_ *G* ^*T*^ = −305.0 kJ/mol. Δ_*r*_ *G* ^*T*^ was subsequently corrected for the concentration of the reactants (using Eq. 6) to obtain Δ_*r*_ *G* ^*T,c*^, the Gibbs energy of the reaction at process conditions. The concentration of the reactants changes with the chosen fermentation parameters such as gas flow and gas recycle rate. However, due to the low CO, but high CO_2_ concentration in the fermentation broth, the absolute value of Δ_*r*_ *G* ^*T,c*^ is lower than that of Δ_*r*_ *G* ^*T*^.

The Gibbs energy Δ_*r*_ *G* ^*T,c*^ released during product formation is used by the cell mass for maintenance and cell growth [24]. The rate of substrate conversion for product and cell mass formation under the respective process conditions is eventually summarized in the process reaction. Box 1 exemplifies how the process reaction can be derived.

#### Box 1. Process reaction (CO to acetone)

Catabolic reaction (Eqs. 3, 4); in mol: $- 8\,{\text{CO}} - 3\,{\text{H}}_{ 2} {\text{O}} + 1\,{\text{C}}_{ 3} {\text{H}}_{ 6} {\text{O}} + 5\,{\text{CO}}_{ 2} ;$

$$\Delta_{r} G^{0} = - 322.8\,{\text{kJ}}/{\text{mol}};\quad \Delta_{r} H^{0} = - 475.5\,{\text{kJ}}/{\text{mol}}$$

Correction for process temperature *T* = 333.15 K (Eq. 5):

$$\begin{aligned} \Delta_{r} G^{T} &= \Delta_{r} G^{0} \cdot \left( {T/298.15\,{\text{K}}} \right) + \Delta_{r} H^{0} \cdot (1 - T/298.15\, {\text{K}}) \hfill \\ &= - 322.8{\text{ kJ/mol}} \cdot \left( {333.15{\text{ K}} / 298.15{\text{ K}}} \right) + \left( { - 475.5{\text{ kJ/mol}}} \right) \cdot \left( {1 - 333.15{\text{ K}} / 298.15{\text{ K}}} \right) \hfill \\ &= - 305.0{\text{ kJ/mol}} \hfill \\ \end{aligned}.$$

Correction for concentration of the reactants according to Eq. 6; for *c*_acetone_ = 0.50 M; *c*_CO_ = 0.001 M; $c_{{{\text{CO}}_{ 2} }}$ = 0.003 M

$$\begin{aligned} \Delta_{r} G^{T,c} &= \Delta_{r} G^{T} + R \cdot T \cdot \ln \left( {c_{i}^{{\nu_{i} }} } \right) \cdot 10^{ - 3} \hfill \\ &= - 305.0{\text{ kJ/mol}} + 8.3145{\text{ J/K/mol}} \cdot 333.15{\text{ K}} \cdot \ln \left( {0.50^{1} \cdot 0.001^{ - 8} \cdot 0.003^{5} } \right) \cdot 10^{ - 3} \hfill \\ &= - 269.7{\text{ kJ/mol}} \hfill \\ \end{aligned}$$

Gibbs free energy per mol substrate: $\Delta G_{\text{CO}} = ( - 269.7\,{\text{kJ}}/{\text{mol}})/8 = 33.71\,{\text{kJ}}/{\text{mol}} .$ Anabolic reaction (1); in mol: $- 2.1\,{\text{CO}} - 0.60\,{\text{H}}_{ 2} {\text{O}} - 0.20\,{\text{NH}}_{4}^{ + } + 1.0\,{\text{CH}}_{1.8} {\text{O}}_{ 0. 5} {\text{N}}_{ 0. 2} + 1.1\,{\text{CO}}_{ 2} + 0.20\,{\text{H}}^{ + } .$ Maintenance energy requirement: *m*_G_ = 62 kJ/C-mol/h; ratio of maintenance energy requirement and Gibbs free energy per mol substrate: m_G_/Δ*G*_CO_ = 1.8; maintenance reaction (2): catabolic reaction to maintain 1 mol of cell mass per hour; in mol/h: $- { 1}. 8 {\text{ CO }} - \, 0. 6 8 {\text{ H}}_{ 2} {\text{O }} + \, 0. 2 3 {\text{ C}}_{ 3} {\text{H}}_{ 6} {\text{O }} + { 1}. 1 3 {\text{ CO}}_{ 2} + { 1}. 1\cdot 10 2 {\text{ kJ}}/{\text{h}}.$ Growth energy requirement: *a*_G_ = 1000 kJ/C-mol; ratio of growth energy requirement and Gibbs free energy per mol substrate: a_G_/Δ*G*_CO_ = 29.66. Catabolic reaction to provide energy to grow 1 C-mol of cell mass (3); in mol: $- { 29}. 66 {\text{ CO }} - { 11}. 1 2 {\text{ H}}_{ 2} {\text{O }} + { 3}. 71 {\text{ C}}_{ 3} {\text{H}}_{ 6} {\text{O }} + { 18}.54 {\text{ CO}}_{ 2} + {\text{ 1759 kJ}}.$ Growth reaction: combination of anabolic (1) and catabolic reaction (3) for growth of 1 mol cell mass (4); in mol: $- {\text{ 32 CO }} - {\text{ 12 H}}_{ 2} {\text{O }} - \, 0. 20{\text{ NH}}_{4}^{ + } + { 1}.0{\text{ CH}}_{ 1. 8} {\text{O}}_{0. 5} {\text{N}}_{0. 2} + { 3}. 7 1 {\text{ C}}_{ 3} {\text{H}}_{ 6} {\text{O }} + { 19}. 6 4 {\text{ CO}}_{ 2} + \, 0. 20{\text{ H}}^{ + } + 1. 8\cdot 10^{ 2} {\text{kJ}}$ Process reaction (with *μ* = 0.10 h^−1^) according to Eq. 8: Combination of maintenance reaction (2) and growth reaction (4); *q*_*i*_-rates in mol/h: $$\begin{aligned} &( - 1. 8- 3 2\cdot 0. 10){\text{ CO}} + ( - 0. 6 8- 1 2\cdot 0. 10){\text{ H}}_{ 2} {\text{O}} + ( - 0. 2\cdot 0. 10){\text{ NH}}_{4}^{ + } + 1\cdot 0. 10{\text{ CH}}_{ 1. 8} {\text{O}}_{0. 5} {\text{N}}_{0. 2} \hfill \\ & + (0. 2 3+ 0. 10 \cdot 3. 7 1){\text{ C}}_{ 3} {\text{H}}_{ 6} {\text{O}} + ( 1. 1 3+ 1 9. 6 4\cdot 0. 10){\text{ CO}}_{ 2} + (0. 2\cdot 0. 10){\text{ H}}^{ + } \hfill \\ &+ ( 1. 4\cdot 10^{ 2} + 1. 8\cdot 10^{ 2} \cdot 0. 10){\text{ kJ}}/{\text{h}} \hfill \\ &= - 5.0{\text{ CO}} - 1. 9 {\text{ H}}_{ 2} {\text{O}} - 0.0 2 {\text{ NH}}_{4}^{ + } + 0. 10{\text{ CH}}_{ 1. 8} {\text{O}}_{0. 5} {\text{N}}_{0. 2} + 0. 60{\text{ C}}_{ 3} {\text{H}}_{ 6} {\text{O}} + 3. 1 {\text{ CO}}_{ 2} + 0.0 2 {\text{ H}}^{ + } + 1. 6\cdot 10^{ 2} \,{\text{kJ}}/{\text{h}}. \end{aligned}$$ Process reaction per mol of acetone; in mol/h: $- 8. 3 {\text{ CO}} - 3. 2 {\text{ H}}_{ 2} {\text{O}} - 0.0 3 {\text{ NH}}_{4}^{ + } + 0. 1 7 {\text{ CH}}_{ 1. 8} {\text{O}}_{0. 5} {\text{N}}_{0. 2} + 1.0{\text{ C}}_{ 3} {\text{H}}_{ 6} {\text{O}} + 5. 2 {\text{ CO}}_{ 2} + 0.0 3 {\text{ H}}^{ + } + 2. 7\cdot 10^{ 2} {\text{ kJ}}/{\text{h}}.$

### Bioreactor considerations

The given reactor size, the operating pressure and temperature, and the syngas flow rate determine the transfer capacity of the gases into the broth. The amount of gas, which is available to the cell as substrate, is restricted by the low solubility of the gases. Hence, the gas–liquid mass transfer most likely becomes the rate-limiting step of the syngas-to-acetone conversion. As shown in Fig. 1, the gas at the molar flow rate *R*_in_ (in mol/h) is compressed into the bioreactor at the bottom of the reactor, at pressure *p*_b_. The *k*_L_ *a* for CO was determined (using Eqs. 12–15) and the gas transfer rate of CO into the liquid was calculated using Eq. 17 under the assumption that the CO concentration is kept low by the constant uptake by the production host, and it was therefore estimated to be 1% of c*(CO). The concentration of CO_2_ in the liquid phase was calculated using Eq. 18, with the CO_2_ production rate from the process reaction. The rate at which CO enters the liquid phase was obtained by multiplying the CO transfer rate TR(CO) with the liquid volume *V*_liq_. Box 2 shows an example of how the transfer rate of CO is calculated.

The gas leaving the bioreactor consists of gas which was not absorbed into the liquid phase and of CO_2_, which is produced by *M. thermoacetica* during the conversion of CO to acetone. Additionally, the off-gas contains the produced acetone and water. Acetone and water are removed from the off-gas in a condensation step. Acetone is separated from the water in a subsequent distillation step.

We accounted for the loss of product when determining the number of reactors required to meet the desired hourly production (8% of the product is lost in the downstream processes). Additional product losses which occur in the steps from the purified to the final shipped products, for example during packaging, were neglected. The product recovery was simulated as described in the methods section. In our simulation, the water separated from the acetone was recycled to the reactor.

The off-gas from the condensation/distillation step, consisting of H_2_, CO_2_, and CO, can be mixed with fresh syngas and recycled to the reactor. The choice of the recycle rate (as percentage of *R*_in_) is a trade-off between the production rate and the utility costs for gas compression on the one hand, and costs for fresh syngas on the other hand, and will be addressed in the next section.

The gas transfer rate (TR) is dependent on two terms: the volumetric mass transfer coefficient *k*_L_ *a*, and the concentration of the gas in the liquid, *c*_liq_. The *k*_L_ *a*-term is dependent on the average superficial gas velocity, determined by the average gas flow rate and pressure. The *c*_liq_-term, however, is dependent on the partial pressure of the gas going into the reactor (*c*_g_), which is in turn determined by the gas transfer rate TR if the gas is, at least partly, recycled. Therefore, the composition of the gas injected into the bioreactor changes with every recycling round and converges to a steady state for a set recycle rate. Additional file 1: Figure S2 illustrates how the fermentation parameters and terms related to the gas–liquid mass transfer influence each other.

Depending on the rate of gas recycling, the off-gas, which will be purged, contains a certain amount of CO. Since CO is considered as a pollutant [57], the CO emission of the production process has to be limited. Cost of measures, such as flaring [58], was not taken into account in this study.

#### Box 2. Calculation of the CO transfer rate

For *R*_in_ = 8·10^5^ mol/h; *R*_out_ = 7·10^5^ mol/h; *p*_b_ = 3.5·10^5^ Pa; *p* = 2·10^5^ Pa; *A* = 28 m^2^; c*(CO) = 1 mol/m^3^; Calculation of the pressure-corrected gas flow (Eq. 12):

$$F_{\text{av}} [{\text{m}}^{3} /{\text{h}}] = \left[ {\left( {R_{\text{in}} + R_{\text{out}} } \right) \cdot 0.5 \cdot R \cdot T} \right]/p = 10.4 \cdot 10^{3} \,{\text{m}}^{3} /{\text{h}}$$

Calculation of the superficial gas velocity (Eq. 13):

$$v_{\text{gs}}^{\text{c}} = F_{\text{av}} /A = 10. 4\cdot 10^{ 3} {\text{m}}^{ 3} /{\text{h}}/ 2 8 {\text{ m}}^{ 2} = {\text{ 371 m}}/{\text{h }} = \, 0. 10 3 {\text{ m}}/{\text{s}}$$

Calculation of the volumetric mass transfer coefficient (Eqs. 14, 15):

$$\begin{aligned} k_{\text{L}} a &= 0.32 \cdot (D_{\text{CO}} /D_{{{\text{O}}_{ 2} }} ) \cdot \left( { v_{\text{gs}}^{\text{c}} } \right)^{0.7} \cdot \theta^{{T - 293.15\,{\text{K}}}} \hfill \\ &= 0. 3 2\cdot \left( { 2.0 8\cdot 10^{ - 5} / 2. 1 5\cdot 10^{ - 5} } \right) \cdot 0. 10 3^{0. 7} \cdot 1.0 2 2^{ 3 3 3. 1 5- 2 9 8. 1 5} \hfill \\ &= 0. 1 3 5\,{\text{s}}^{ - 1} = 4 8 6\,{\text{h}}^{ - 1} \hfill \\ \end{aligned}$$

Calculation of the CO transfer rate (Eq. 17):

$${\text{TR(CO)}} = k_{\text{L}} a \cdot \left( {c^{*} - c_{\text{l}} } \right) = k_{\text{L}} a \cdot 0.99 \cdot c^{*} = 481\,{\text{mol/m}}^{ 3} / {\text{h}}$$

### Parameters for plant optimization

Because the system is considered in steady state, all the CO which enters the liquid phase, *R*_liq_(CO), will be converted by the cell mass. In our fermentation set up, the reactor size (30 m height, 6 m diameter) and the composition of the syngas are fixed. Additionally, the pressure-corrected superficial gas velocity $v_{\text{gs}}^{\text{c}}$ was kept below 0.15 m/s. The molar flow rate of the gas into the reactor, *R*_in_, and the ratio of recycled gas, *R*_rec_, could be varied. However, several optimization constraints restrict the choice of *R*_in_ and *R*_rec_ in an industrial setting:

#### Concentration of acetone in liquid

The concentration of acetone in the fermentation broth was determined using Eqs. 32 and 33, assuming steady state: acetone leaves the reactor with the outflowing gas stream at the same rate as it is produced by the cell mass. Two factors have an effect on the acetone concentration in the fermentation broth: Firstly, the acetone concentration is positively correlated to the production rate, and the production rate decreases with increasing *R*_rec_ values. Secondly, the acetone concentration decreases with higher gas outflow rates (when *R*_in_ high), due to the gas-stripping effect. Hence, the acetone concentration can be kept low when both *R*_in_ and *R*_rec_ are high. Tests in our lab showed that *M. thermoacetica* strain ATCC 39073 can tolerate acetone concentrations up to 30 g/l without being affected in its growth behavior (unpublished data).

#### Number of reactors required to meet the desired production

The more CO is available to the cell mass, the more acetone is produced. This can be achieved by high gas inflow (*R*_in_ high) and low recycle rate (*R*_rec_ low). With increasing acetone production per reactor, fewer reactors are required to achieve the desired acetone production.

#### Variable costs of production

Increasing the acetone production by raising the flow of fresh syngas comes at a cost: the variable costs for feedstock and gasification are rising. Additionally, it has to be taken into account that increasing the gas recycle rate (*R*_rec_/*R*_in_ high) leads to efficient utilization of the substrate. However, a high gas recycling rate increases the number of reactors required to meet the desired production metrics.

### Variable costs of production

The variable costs of syngas production and fermentation are crucial optimization parameters in the process design. The costs can be categorized into pre-fermentation costs (that is syngas production) and fermentation-related costs. As described above, we determined that the cost for syngas was derived from BOF gas, natural gas, and corn stover. Only BOF-derived syngas with a cost of 7.6·10^−4^ $/mol CO is, based on the theoretical conversion yield, an interesting source for syngas to date. As fermentation-related variable costs we took into account the costs for chilled water, the power requirements for gas compression, and product recovery. Other fermentation-related costs, such as media sterilization, disposal of fermentation residue, and media components were not taken into account.

To determine the requirements of chilled water, the heat balance of the reaction was set up, and the rate of chilled water was determined using Eq. 31 and translated into costs (0.05 $/m^3^ chilled water). Box 3 contains examples of how the heat balance was set up and how the cooling requirements can be determined. The power requirements for gas compression were calculated using Eq. 22. Box 4 exemplifies how those power requirements are determined. The power requirements for product recovery (condensation and distillation) were retrieved from simulations with SuperPro Designer^®^ and AspenPlus^®^ and converted into costs assuming 0.08 $/kWh. Further details on the selection of the downstream process scheme are described in the Additional file 2.

#### Box 3. Heat balance and calculation of requirements for chilled water

Net heat balance: $\Delta H_{\text{net}} = \Delta H_{r} + \Delta H_{\text{gas}}^{\text{comp}} + \Delta H_{\text{acetone}}^{\text{evap}} + \Delta H_{\text{water}}^{\text{evap}} .$Heat released by the cell mass per reactor (obtained from the process reaction), e.g. $\Delta H_{r} = - 1.5 \cdot 10^{4} \,{\text{MJ}}/{\text{h;}}$Rate of heat generated by gas compression: $R_{\text{gas}} = 4 \cdot 10^{5} \,{\text{mol/h}}\; (T_{2} { = 430}\,{\text{K;}}\;c_{v} { = 2} . 1 4\cdot 1 0^{ - 2} \,{\text{kJ/(mol}} \cdot {\text{K));}}$

$$\Delta H_{\text{gas}}^{\text{comp}} = 4 \cdot 10^{5} {\text{mol}}/{\text{h}} \cdot 2.14 \cdot 10^{ - 2} {\text{kJ}}/({\text{mol}} \cdot {\text{K}}) \cdot (333\,{\text{K}} - 430\,{\text{K}}) = - 9.4 \cdot 10^{2} {\text{MJ}}/{\text{h}} .$$

Rate of acetone evaporation equals the acetone production rate, e.g. $R_{\text{acetone}}^{\text{vap}} = 3.6 \cdot 10^{4} \,{\text{mol}}/{\text{h}} .$  Rate of water evaporation; e.g. $R_{{{\text{H}}_{ 2} {\text{O}}}}^{\text{vap}} = 1.1 \cdot 10^{5} {\text{mol}}/{\text{h}} .$ Using the heat of vaporization for water and acetone at 60 °C (Additional file 1: Table S13):

$$\Delta H^{\text{vap}} ({\text{H}}_{ 2} {\text{O}}) = 42.6\,{\text{kJ}}/{\text{mol}};\quad \Delta H^{\text{vap}} ({\text{acetone}}) = 29.0\,{\text{kJ}}/{\text{mol}}$$

$$\Delta H_{\text{acetone}}^{\text{evap}} = R_{\text{acetone}}^{\text{vap}} \cdot \Delta H^{\text{vap}} ({\text{acetone}}) = 3.6 \cdot 10^{4} {\text{mol}}/{\text{h}} \cdot 29.0\,{\text{kJ}}/{\text{mol}} = 1.0 \cdot 10^{3} {\text{MJ}}/{\text{h}}$$

$$\Delta H_{\text{water}}^{\text{evap}} = R_{\text{water}}^{\text{vap}} \cdot \Delta H^{\text{vap}} ({\text{water}}) = 1.1 \cdot 10^{5} {\text{mol}}/{\text{h}} \cdot 42.6\,{\text{kJ}}/{\text{mol}} = 4.7 \cdot 10^{3} {\text{MJ}}/{\text{h}}$$

Calculation of the net heat balance:

$$\begin{aligned} \Delta H_{\text{net}} &= \Delta H_{r} + \Delta H_{\text{gas}}^{\text{comp}} + \Delta H_{\text{acetone}}^{\text{evap}} + \Delta H_{\text{water}}^{\text{evap}} \hfill \\ &= ( - 1.5 \cdot 10^{4} - 9.4 \cdot 10^{2} + 1.0 \cdot 10^{3} + 4.7 \cdot 10^{3} ){\text{MJ}}/{\text{h}} &= - 10^{4} \,{\text{MJ}}/{\text{h}} \hfill \\ \end{aligned}$$

Calculation of required amount of cooling water (Eq. 30):

$$R_{\text{chill}} = |\Delta H_{\text{net}} |/(c_{p} \cdot \Delta T) = (10^{10} \,{\text{J}}/{\text{h}})/((71.19\,{\text{J}}/{\text{mol}}/{\text{K}}) \cdot (333 - 277){\text{K}}) = 2.5 \cdot 10^{6} \,{\text{mol}} = 45\,{\text{m}}^{3} /{\text{h}}$$

Costs for cooling water: 45 m^3^/h∙0.05 $/m^3^ = 2.3 $/h

#### Box 4. Calculation of power requirements for gas compression

Power requirement to compress the syngas into the reactor (Eq. 21):

$$P[W] = \frac{\gamma }{\gamma - 1} \cdot p_{1} \cdot V_{1} \cdot \left[ {\left( {\frac{{p_{2} }}{{p_{1} }}} \right)^{(\gamma - 1)/\gamma } - 1} \right] \cdot (100/70)$$

*p*_1_ = 1.0∙10^5^ Pa; for *p*_2_ = *p*_b_ = 3.5∙10^5^ Pa; Compression of 7∙10^3^ m^3^/h; Composition syngas e.g.: CO (81 mol%), CO_2_ (0 mol%), H_2_ (2 mol%); N_2_ (17 mol%)

$$\gamma_{\text{gas}} = \frac{{c_{\text{p}}^{\text{gas}} }}{{c_{\text{v}}^{\text{gas}} }} = \frac{{\sum {y_{i} c_{{{\text{p}},i}} } }}{{\sum {y_{i} c_{{{\text{v}},i}} } }} = 1.40$$

$$\begin{aligned} P[W] &= \frac{{\gamma_{\text{gas}} }}{{\gamma_{\text{gas}} - 1}} \cdot p_{1} \cdot V_{1} \cdot \left[ {\left( {\frac{{p_{2} }}{{p_{1} }}} \right)^{{(\gamma_{\text{gas}} - 1)/\gamma_{\text{gas}} }} - 1} \right] \cdot (100/70) \hfill \\ & = 3.5 \cdot 1.0 \cdot 10^{5\,} \,{\text{Pa}} \cdot \frac{{7 \cdot 10^{3} }}{3600}\,{\text{m}}^{3} /{\text{s}} \cdot \left[ {\left( {\frac{{3.5 \cdot 10^{5} \,{\text{Pa}}}}{{1.0 \cdot 10^{5} \,{\text{Pa}}}}} \right)^{0.29} - 1} \right] \cdot (100/70) = 426\,{\text{kW}} \hfill \\ \end{aligned}$$

### Analysis of a fermentation scenario

We tested process scenarios with BOF gas-derived syngas. *R*_rec_/*R*_in_ combinations were varied to find a process set-up at which the above-mentioned parameters of acetone concentration, plant sizing (number of reactors), and variable costs are within a reasonable range. Here we present the outcome of a production scenario in which the gas flow rate in the reactor (*R*_in_) was set to 6∙10^5^ mol/h. At this gas flow rate the superficial gas velocity $v_{\text{gs}}^{\text{c}}$ (corrected for the average gas flow in the reactor) equals 0.082 m/s. We tested different *R*_rec_/*R*_in_ combinations and their influence on the process parameters. In a scenario where the gas compressed into the reactor contains 20 mol% recycled gas (*R*_rec_ = 1.2∙10^5^ mol/h), the acetone concentration in the broth (21 g/l) stays below the toxicity limit. Our model predicts an hourly biological acetone production of rate 2225 kg/h (concentration of cell mass 1.3 g/l; productivity: 2.29 g/g/h). Under the given process conditions the reactor off-gas has an acetone content of 6 mol%.

We simulated the acetone recovery by condensation and distillation with SuperPro Designer^®^ and AspenPlus^®^. Additional file 1: Table S14 illustrates the composition of the off-gas obtained at the top outlet of the fermenter, which is received by the downstream operations as feedstock. The purity of the final product is 99% and we determined a loss of maximal 8% h^−1^. Accounting for the product recovery loss, 2058 kg final product would be produced per hour in the analyzed scenario. To reach the desired production metrics of 3.79·10^3^, two reactors would be required. For this scenario we determined variable production costs of 0.389 $/kg acetone. The contributions to the costs are: 34.1% for gaseous substrate, 0.3% for chilled water, 21.5% for gas compression, and 44.1% for downstream processing. The utilities for downstream processing are listed in detail in Additional file 1: Tables S15 and S16.

At the presented scenario, the CO-to-acetone conversion reaches 74% of the theoretical carbon yield. To increase the yield, a higher gas recycle rate could be implemented. However, increasing the gas recycle rate would not be beneficial for the number of reactors required to meet the desired production metrics.

## Discussion

### Economic feasibility of acetone production from syngas

In this study, we have analyzed the conversion of syngas to acetone using the hypothetical thermophilic production strain *Moorella thermoacetica* with regard to thermodynamic considerations of the bacterial physiology, to bioreactor design limitations, and to economic feasibility.

We have estimated the costs for syngas with a CO content above 80 mol% derived from three different sources and only BOF gas was identified as an interesting syngas source from an economic perspective. Therefore we have determined the other main variable production cost (gas compression, downstream processing, and chilled water) for a representative production process. Those variable production costs sum up, together with the costs for the gaseous substrate, to 389 $/t.

As mentioned before, off-gas is not utilized in US steel mills to date. Therefore we assume that BOF gas comes free of charge. In Europe, however, only 25% of the BOF gas is flared and the rest is utilized for the generation of electricity and heat [32]. We tested a scenario in which the presented acetone production process would be implemented in a scenario where BOF gas is not underutilized, that is, compensation for the feedstock is required. Assuming an additional cost of 0.0036 $/mol CO for BOF gas (see Additional file 1), would increase the variable production cost to 1018 $/t acetone, which would not lead to a profitable process to date.

Alternative sources for syngas besides those analyzed in this study can be considered. Biogas for example is another source of CH_4_-rich gas which could be reformed to a CO-rich syngas. However, biogas has a significant fraction of CO_2_ [59]. Therefore, an additional acid gas removal step would be required to reach a gas composition of natural gas before reforming. This would add an additional cost to the already high syngas production costs from natural gas of 298 $/t CO. This makes syngas derived from biogas less interesting as CO source for the production process in this study.

### Approach of this study

#### Utilization of H_2_/CO_2_

We assumed that the production organism *M. thermoacetica* would be converting only CO to acetone, since there is no pathway existing to generate net ATP from the conversion of H_2_/CO_2_ to acetone [60]. Although no net ATP is generated, alternative metabolic reactions would allow H_2_/CO_2_ to serve as substrate: firstly, acetate could be generated as byproduct. The second alternative would require that conversion of CO to acetone would deliver the energy required for cell maintenance and growth. The latter scenario could be realized by metabolic engineering strategies to ensure metabolization of H_2_/CO_2_ with net ATP generation. However, shifting the composition of the biomass-derived syngas towards CO using rWGS reaction is a minor contributor to the overall production costs, meaning the benefit of engineered H_2_/CO_2_ utilization would be small. However, conversion of CO results in the production of a certain amount of CO_2_: for the production of acetone, 0.625 mol CO_2_ is produced per mol converted CO. CO_2_ is diluting the off-gas considerably, thereby making the gas recycling less effective. An option would be the removal of CO_2_ from the off-gas. Several techniques for CO_2_ capture from gases are described [61].

#### Thermodynamics approach

The approach of using the principles of thermodynamics to estimate the conversion rate has to be used with caution for acetogenic bacteria. The metabolism of acetogens is known to perform close to thermodynamic limits [11], and process conditions (reactant concentration, pressure, temperature) might have a disproportionately high impact on the estimated free energy of the product reaction. Therefore, erroneous assumptions can have significant impact on the outcome of the study.

The thermodynamics approach is based on the energy requirements for cell maintenance, and no accurate values have been reported in literature for *M. thermoacetica*. In the metabolic model published in 2015, a maintenance requirement of 0.12 mmol ATP/g/h was used [12]. With around 46.2 kJ energy conserved per mol ATP for homoacetogenic bacteria [62], that would equal 5.5·10^−3^ kJ/g/h (0.14 kJ/C-mol/h assuming 24.6 g/C-mol), which seems a surprisingly low value compared to the 62 kJ/C-mol/h used in this study. The non-growth associated maintenance ATP requirement for *E. coli,* as comparison, is reported to be 8.39 mmol ATP/g/h [63]. Acquiring more accurate values for the maintenance energy requirement from experimental data would increase the accuracy of our model. Since suboptimal culturing conditions increase the maintenance energy requirement [40], it is relevant to retrieve the data under fermentation conditions that resemble an industrial set-up.

From the data generated with our model, the CO uptake rates can be determined. The CO uptake rate is around 323 mmol CO/g/h for the production scenario presented. This value is relatively high when compared to CO uptake rates described for acetogens in literature [12, 64, 65]. A possible reason is a difference in the growth rate. In this study, we assumed a growth rate of 0.1 h^−1^ (as published by Kerby and Zeikus [55]). When assuming a growth rate of 0.01 h^−1^ (as described by Islam et al. for growth on CO [12]), the uptake rate predicted with our model decreases to 141 mmol CO/g/h. Another reason for potentially overestimating the CO uptake rate can be the maintenance energy requirement, which might be lower than the estimated 62 kJ/C-mol/h (as discussed above). When lowering the maintenance energy requirement to 20 kJ/C-mol/h (with µ = 0.01 g/g/h), the average uptake rate decreases to 59 mmol CO/g cell mass. Additionally, it is reported that high concentrations of dissolved CO are inhibitory for acetogens, and that the process is at a certain gas supply rate biologically limited instead of gas transfer limited [65]. However, the influence of changes to our model which result in lower CO uptake rates have minor impact on the outcome of our analysis regarding production cost and plant sizing.

In 2015, Chen et al. published a spatiotemporal metabolic model for bubble column reactors with the acetogen *C. ljungdahlii*, in which model iHN637 was integrated [66], and a similar approach could be applied to perform an economic analysis for the process presented in this study. However, integration of model iAI558 of *M. thermoacetica*, in which for example a novel mechanism of energy conservation was implemented [12], would have based the study on different assumptions regarding the metabolism of the production strain. Future implementation of an updated version of iAI558 including the acetone pathway would nonetheless be possible.

#### Reactor design

Traditionally, continuous stirred-tank reactors (CSTR) are employed in syngas fermentation. Stirring breaks the gas bubbles and thereby increases the interfacial area and the gas retention time [67]. However, stirring increases the power usage. An alternative, suitable for industrial applications, are bubble column reactors [67], which we chose for this study. More sophisticated bioreactor set-ups that increase the gas–liquid mass transfer could further improve the yield. This could for example be achieved with microbubble dispersion stirred-tank reactors. Microbubbles, which have an average diameter of only 50 µm compared to the normal 3–5 mm bubble diameter, offer a significantly higher gas–liquid interfacial area [68], but generation of microbubbles will also require extra energy and costs. Biofilm reactors are another option, and can result in an increased interfacial area between substrate and the production host. *M. thermoacetica* is reported to be capable of forming thin biofilms [69].

To determine the requirement for chilled water, only fermentation-related processes (heat generated by the cell mass, evaporating water and acetone, heat released during adiabatic compression) were taken into account. Other energy requirements, which for example arise during syngas production or product recovery, were accounted for when determining the utilities.

Additionally, the variable costs of production which do not occur continuously, such as sterilization, costs for media components, and disposal of the acetone loss, were omitted. However, this study is intended to serve as a preliminary feasibility analysis, with a focus on variable costs of production as the main criterion for an economically viable process. In a more elaborate model an overall integrated heat balance, a more comprehensive overview of the variable production costs as well as fixed operating costs and capital costs could be implemented.

## Conclusions

In this study, we have analyzed the feasibility of acetone production from syngas from three different sources using the thermophilic acetogen *M. thermoacetica* as a hypothetical production host with regard to metabolic and economic aspects.

Syngas contains H_2_, CO_2_, and CO as potential substrate. However, when acetone is the sole end product, ATP is only generated when CO is used as substrate. We have determined the costs for syngas with a CO content higher than 81 mol% from BOF gas, from natural gas, and from biomass. We identified syngas derived from BOF gas as the only syngas source to date which is economically promising for the production of acetone.

For different fermentation scenarios with varying gas feed and gas recycle rates, we analyzed the variable cost of production and the cost contribution of the single process steps, the number of reactors required to produce at the desired rate of 30 kt/year, the efficiency of the gas utilization, and parameters related to cell mass and productivity. This was done by setting up the process reaction in which the rate of acetone formation from CO under the process conditions is described. The amount of available substrate was determined by the rate of CO transferred into the fermentation broth, in turn depending on the chosen process parameters.

We presented data for a representative fermentation scenario in which 6∙10^5^ mol/h gas, containing 4.8∙10^5^ mol/h syngas derived from BOF gas and 1.2∙10^5^ mol/h recycled off-gas, is fed in a bubble column and converted to acetone by *M. thermoacetica* at 60 °C. The variable production costs comprising the cost for syngas, gas compression, chilled water, and product recovery were determined to be 389 $/t, with the cost for syngas as the main contributor.

Here, we have illustrated an application of the thermodynamics approach, in which the rate of acetone production is derived from the Gibbs energy of product formation, the maintenance and growth energy requirements, and the growth rate, for the formation of a volatile compound from a gaseous substrate. As the approach is based on certain assumptions, such as the maintenance energy requirement, experimental data would increase the accuracy of our model. Since the heterologous expression of the acetone pathway in *M. thermoacetica* has not been reported so far, the study is based on a hypothetical production strain. We hope that further development of the genetic toolbox for *M. thermoacetica* or similar thermophilic acetogens will soon make heterologous acetone pathway expression possible, since this will enable experimental studies at reactor scale.

This study exemplifies the importance of a metabolic feasibility analysis and we encourage other researchers to apply the presented approach to other bioproduction scenarios in order to estimate the economic viability of the process and to obtain insights into potential bottlenecks.

## Abbreviations

*A*: cross-sectional area of reactor; in m^2^; *a*_*G*_: energy requirement for growth of 1 mol cell mass; in kJ/C-mol; ATP: adenosine triphosphate; BOF: basic oxygen furnace; *c*_CM_: concentration of cell mass; in C-mol/m^3^; *c*_g_: concentration in gas phase; *c*_*i*_: concentration of compound *i*; *c*_liq_: concentration in liquid phase; *c*_p_: specific molar heat capacity at constant pressure; in kJ/mol/K; *c*_v_: specific molar heat capacity at constant volume; in kJ/mol/K; c*: dissolved gas concentration at equilibrium; in mol/m^3^; *D* ^0^: standard diffusion coefficient; in cm^2^/s; *D*_*i*_: diffusion coefficient; in m^2^/h; *F*_av_: average gas flow rate; in m^3^/h; *F*_*out*_: flow rate of gas leaving the reactor; in m^3^/h; *g*: gravitational constant; in m/s^2^; *h*: height; in m; *H* ^0^: Henry’s law solubility constant at standard temperature 298.15 K; in mol/kg/bar; *H*_T_: Henry’s law solubility constant corrected for temperature *T*; in mol/m^3^/Pa; *k*: temperature correction factor for Henry’s law constant; *k*_L_ *a*: volumetric mass transfer coefficient; in s^−1^; *m*_G_: maintenance energy requirement for 1 mol cell mass; in kJ/C-mol/h; *n*_CM_: molar amount of cell mass; in C-mol; *p*: logarithmic mean pressure in the reactor vessel; in Pa; *p*_b_: pressure at bottom of reactor; in Pa; *p*_*i*_: partial pressure of compound *i*; in Pa; $p_{i}^{\text{vap}}$: vapor pressure; in Pa; *p*_*t*_: pressure at top of reactor; in Pa; *q*_heat_: cell mass-specific rate of heat production; *q*_*i*_: cell mass-specific rate of production or consumption of reactant *i*; *R*: gas constant; 8.314 (m^3^ Pa)/K/mol; *R*_chill_: rate of chilled water; in mol/h; *R*_gas_: rate of syngas inflow; in mol/h; *R*_in_: rate of gas inflow; in mol/h; *R*_liq_: rate of transfer from gas to liquid phase; in mol/h; *R*_out_: rate of gas outflow; in mol/h; *R*_p_: rate of production; in mol/h; *R*_rec_: rate of recycled gas inflow; in mol/h; rWGS: reverse water–gas shift reaction; *T*: temperature; TR: gas transfer rate; in mol/m^3^/h; $v_{\text{gs}}^{\text{c}}$: pressure-corrected superficial gas velocity; *V*_liq_: volume broth; in m^3^; *V*_reactor_: volume reactor; in m^3^; WLP: Wood–Ljungdahl pathway; *y*: mol-fraction of the gas; Δ_*f*_ *G*_*i*_^0^: Gibbs energy of formation of compound *i* at standard conditions (*T* = 298.15 K); in kJ/mol; Δ_*f*_ *H*_*i*_^0^: heat formation of compound *i* at standard conditions (*T* = 298.15 K); in kJ/mol; Δ*H* ^comp^: rate of heat released by gas compression; in kJ/h; Δ*H* ^evap^: rate of vaporization heat; in kJ/h; Δ*H*_growth_: rate of heat released by growth reaction; in kJ/h; Δ*H*_main_: rate of heat released by maintenance reaction; in kJ/h; $\Delta H_{i}^{\text{vap}}$: heat of vaporization for compounds *i*; in kJ/mol; *Δ*_*r*_ *G* ^*0*^: Gibbs energy of a reaction at standard conditions (*T* = 298.15 K and *c*_*i*_ = 1 M); in kJ/mol; Δ_*r*_ *G* ^*T*^: Gibbs energy of a reaction at process temperature *T;* in kJ/mol; *Δ*_*r*_ *G* ^*T,c*^: Gibbs energy of a reaction corrected for process temperature and concentration of reactants; in kJ/mol; Δ_*r*_ *H* ^0^: enthalpy of reaction at standard conditions; in kJ/mol.

### Greek letters

*ε*: holdup of reactor; *γ*: ratio of specific molar heat capacity at constant pressure and at constant volume; *µ*: growth rate; in h^−1^; *µ* ^0^: dynamic viscosity at 298.15 K; *ν*_*i*_: stoichiometric coefficient of reactant *i*; $v_{i}^{\text{growth}}$: stoichiometric coefficient of reactant *i* in growth reaction;$v_{i}^{\text{main}}$: stoichiometric coefficient of reactant *i* in maintenance reaction; *ρ*: density; in kg/m^3^; *θ*: correction factor to calculate *k*_L_ *a* at process temperature.

### Units

atm: atmosphere; J: Joule; K: Kelvin; m: meter; M: “molar”, 1 M = 1 mol/liter; °C: degree Celsius; Pa: Pascal; t: tons, 10^3^ kg.

## Additional files

**Additional file 1.** Additional information, including Tables S1–S16; Figures S1, S2; derivation of Eq. 15; calculation of electricity generation with BOF gas.

**Additional file 2.** Selection of downstream process scheme.
