# Supplementary material for: Thermodynamics and economic feasibility of acetone production from syngas using the thermophilic production host Moorella thermoacetica
Source: Biotechnol Biofuels. 2017 Jun 12;10:150. doi: 10.1186/s13068-017-0827-8 (PMC5469130; doi:10.1186/s13068-017-0827-8)
Supplement: Supplementary file 1 — Additional file 1. Additional information, including Tables S1–S16; Figures S1, S2; derivation of Eq. 15; calculation of electricity generation with BOF gas. [file 13068_2017_827_MOESM1_ESM.docx]

Supplementary INFORMATION 1

Tables

**Table S 1.** Composition of BOF gas before and after acid gas removal. According to [1]. Temperature of the gas after acid gas removal according to [2].

|  | **BOF**  in vol% | **After acid gas removal**  in vol% | **Concentration**  in mol/m^3^  (1 atm; 297 K) |
| --- | --- | --- | --- |
| CO | 70 | 81 | 33.2 |
| H_2_ | 2 | 2 | 0.819 |
| N_2_ | 15 | 17 | 6.96 |
| CO_2_ | 13 | - | - |

**Table S 2.** Costs for acid gas removal of BOF gas. Density of CO: 1.165 kg/m^3^.

| **Utility** | **Per 1,000 m^3^ feedstock**  **(810 m^3^ CO out)** | **Cost for utility** | **Reference** |
| --- | --- | --- | --- |
| HP Steam | 0.8 t | 30 $/t | [1, 3] |
| Electricity | 18 kWh | 0.08 $/kWh | [4] |
| Solvent | (0.2 $) | 0.2 $ | [1] |
| total |  | 25.64 $ = 0.0317 $/m^3^ CO = 27 $/t CO |  |

**Table S 3.** Summary of natural gas reforming. Cost of reforming steam based on [5].

| **Cost Contribution** | **$/t CO** |
| --- | --- |
| Feedstock | 145.92 |
| Reforming | 20.85 |
| rWGS (see table S4) | 131.66 |
| Total | **298.43** |

**Table S 4.** Summary of energy consumption of rWGS [3]; Output: 446 kg/h CO.

| **Utility Consumption** | kWh | GJ | $/GJ | cost ($) |
| --- | --- | --- | --- | --- |
| Chilled water | 501.903 | 1.807 | 1 | 1.81 |
| Fuel | 460.431 | 1.658 | 20 | 33.15 |
| Refrigerant | 347.431 | 1.251 | 19 | 23.76 |
| Total |  |  |  | **58.72** |

**Table S 5.** Composition of syngas derived from corn stover after rWGS and drying.

|  | **Composition by mass**  in % | **Composition**  in mol% | **Concentration**  in mol/m^3^ |
| --- | --- | --- | --- |
| CO | 83.75% | 86.32% | 38 |
| H_2_ | 0.19% | 2.70% | 1.2 |
| CO_2_ | 15.59% | 10.22% | 4.5 |
| H_2_O | 0.47% | 0.76% | 0.34 |

**Table S 6.** Costs for corn stover feedstock, logistics and preprocessing (on feedstock basis).

|  |  | **Reference** | **Cost**  **[$/t]** |
| --- | --- | --- | --- |
| **Feedstock** | Profit | [6] | 29.28 |
|  |  |  | **29.28** |
| **Logistics** | Harvest | [7] | 40.38 |
|  | Land | [7] | 18.15 |
|  | Loading and unloading | [7] | 6.94 |
|  | Transportation | [7] | 21.98 |
|  |  |  | **87.46** |
| **Feedstock preprocessing** | Grinding | [8] | 10.00 |
|  | Briquetting | [8] | 12.50 |
|  |  |  | **22.50** |
|  | Total |  | **139.2** |

**Table S 7.** Detailed costs for gasification of preprocessed biomass and cleaning of gas on feedstock basis. Capacity: 83.33 t/h; Price electricity: 0.08 $/kW. 52% of the preprocessed feedstock (by mass) is retained as syngas and impurities. Therefore, from 1 t of corn stover briquettes, 520 kg syngas with impurities are obtained. The mol% of the single components is known. Reference: [9].

|  |  | **Power usage**  in MW/h] | **Power usage**  in kW/t | **Cost**  in $/t |
| --- | --- | --- | --- | --- |
| **Gasification and cleaning** | Lock hopper | 0.18 | 2.16 | 0.17 |
|  | Acid gas removal | 1.59 | 19.08 | 1.53 |
|  | Air separation unit (ASU) | 6.31 | 75.72 | 6.06 |
|  | Oxygen compressor (OSU) | 2.80 | 22.6 | 2.69 |
|  | Total |  |  | **10.44** |

**Table S 8.** Cost for gas reforming (rWGS, drying). Price electricity: 0.08 $/kW. Reference: [3].

|  | **Power usage**  in MW/h | **Cost**  in $/h |
| --- | --- | --- |
| **Gas reforming** | 1.69 | 135.22 $ |

**Table S 9.** Standard molar Gibbs energy of formation and standard molar enthalpy (heat) of formation in kJ/mol at 298.15 K.

| **Compound** | **State** | **Formula** | **∆*_f_G^0^***  in kJ/mol | | **∆*_f_H^0^***  in kJ/mol | |
| --- | --- | --- | --- | --- | --- | --- |
| Acetone | *liquid* | C_3_H_6_O | -159.7 | [10] | -248.4 | [11] |
| Carbon dioxide | *gaseous* | CO_2_ | -394.4 | [11] | -393.5 | [11] |
| Carbon monoxide | *gaseous* | CO | -137.2 | [11] | -110.5 | [11] |
| Water | *liquid* | H_2_O | -237.1 | [11] | -285.8 | [11] |

**Table S 10.** Coefficients used for calculation of the gas-liquid transfer rates.

| Diffusivity coefficient; in cm^2^/s | *D^0^*(CO_2_) | 1.92·10^-5^ | [12] |
| --- | --- | --- | --- |
|  | *D^0^*(CO) | 2.03·10^-5^ | [12] |
|  | *D^0^*(O_2_) | 2.10·10^-5^ | [12] |
| Dynamic viscosity at 298.15 K; in cm^2^/s | *µ^0^*(CO_2_) | 1.50·10^-5^ | [13] |
|  | *µ^0^*(CO) | 1.77·10^-5^ | [13] |
|  | *µ^0^*(O_2_) | 2.05·10^-5^ | [13] |
| Dynamic viscosity at 333.15 K; in cm^2^/s | *µ*(CO_2_) | 1.67·10^-5^ | [13] |
|  | *µ*(CO) | 1.93·10^-5^ | [13] |
|  | *µ*(O_2_) | 2.24·10^-5^ | [13] |
| Henry's law solubility; in mol/kg/bar | *H^0^*(CO_2_) | 3.50·10^-2^ | [14] |
|  | *H^0^*(CO) | 9.90·10^-4^ | [14] |
|  | *H^0^*(acetone) | 30 | [14] |
| Henry's law solubility temperature correction factor; in K | *k*(CO_2_) | 2,400 | [14] |
|  | *k*(CO) | 1,300 | [14] |
|  | *k*(acetone) | 4,600 | [14] |

**Table S 11.** Specific molar heat capacity at constant pressure and ratio to specific molar heat capacity at constant volume.

| Specific molar heat capacity at constant pressure; in kJ/(mol·K) | *c_p,CO_* | 2.92·10^-2^ | [14] |
| --- | --- | --- | --- |
|  | *c_p,CO2_* | 3.71·10^-2^ | [14] |
|  | *c_p,H2_* | 2.88·10^-2^ | [14] |
|  | *c_p,N2_* | 2.91·10^-2^ | [14] |
| Ratio of specific heats *γ* = *c_p_ /c_v_* | *ɣ_CO_* | 1.402 | [15] |
|  | *ɣ_CO2_* | 1.299 | [15] |
|  | *ɣ_H2_* | 1.407 | [15] |
|  | *ɣ_N2_* | 1.402 | [15] |

**Table S 12.** Summary of process configurations for simulation of distillation 1 and distillation 2.

| **Column conditions** | **Distillation 1** | **Distillation 2** |
| --- | --- | --- |
| Property method | UNIQUAC | UNIQUAC |
| Number of Stages | 10 | 13 |
| Feed Stage | 4 | 11 |
| Reflux ratio | 0.05 | 1.82 |
| Condenser Pressure (atm) | 1 | 0.5 |
| Condenser Temperature (°C) | -18 | 37 |
| Condenser Utility | Refrigerant | Cooling water |
| Reboiler Pressure (atm) | 2 | 1.5 |
| Reboiler Temperature (°C) | 89 | 109 |
| Reboiler Utility | Low pressure steam | |

**Table S 13.** Coefficients used to set up the heat balance of the fermentation.

| Heat of vaporization at 333.15 K; in kJ/mol | *∆H^vap^*(H_2_O) | 42.6 | [16] |
| --- | --- | --- | --- |
|  | *∆H^vap^*(acetone) | 29.0 | [16] |
| Vapor pressure at 333.15 K; in kPa | *p^vap^*(H_2_O) | 19.9 | [16] |
| Heat capacity at 277.26 K, in J/mol/K | *c_p_*(H_2_O) | 71.19 | [16] |

**Table S 14.** Composition of the fermenter off-gas for the production scenario presented in the study (R_in_ = 6∙10^5^ mol/h; 20 mol% recycled gas).

| **Compound** | **Flowrates in kg/h** |
| --- | --- |
| Acetone | 2,225 |
| CO_2_ | 11,486 |
| CO | 2,763 |
| H_2_ | 26 |
| N_2_ | 3,045 |
| H_2_O | 2,283 |

**Table S 15.** Contribution of utilities to costs of downstream processing. Data for flowrates presented in table S14.

| **Utility Contributions** | | **in kW** | **Utility type** | **Cost in $** |
| --- | --- | --- | --- | --- |
| Condensation | Off-gas compression | 2,705 | Electricity | 216 |
|  | Off-gas cooler | 5,141 | Chilled water | 93 |
|  | Condenser column | 411 | Chilled water | 7 |
| Distillation 1 | Condenser | 27 | Refrigerant | 2 |
|  | Reboiler | 888 | Steam (low pressure) | 18 |
| Distillation 2 | Condenser | 906 | Cooling water | 3 |
|  | Reboiler | 666 | Steam (low pressure) | 14 |
| **Total** | | 10,744 |  | **353** |

Table S 16. Utility costs

| **Utility type** | **Utility cost in $/kW** |
| --- | --- |
| Electricity | 0.08 |
| Refrigerant | 0.068 |
| Chilled water | 0.018 |
| Cooling water | 0.0036 |
| Steam (low pressure) | 0.021 |

Figures


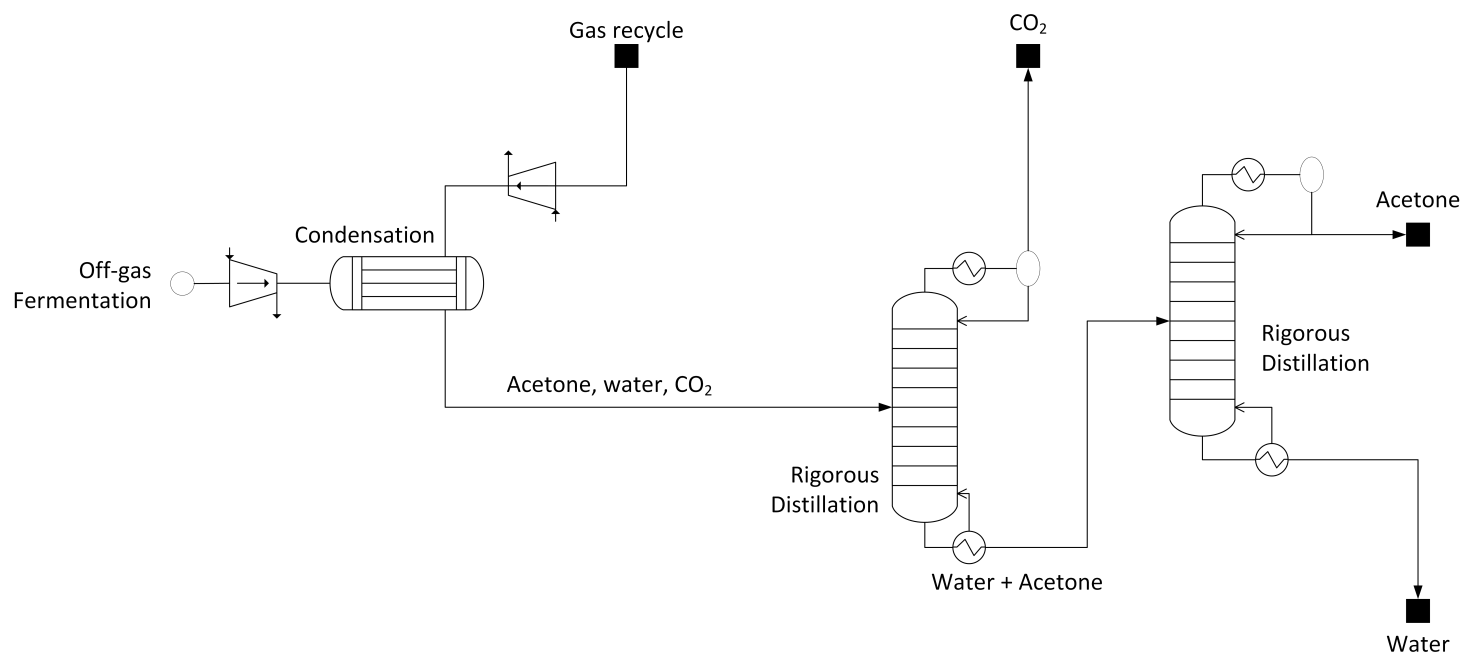


**Figure S1.** Process flow diagram of downstream processing.

**Figure S2**. Overview how coefficients of gas–liquid mass transfer calculations influence each other.

Other

**Derivation of eq. 15 to calculate *kLa(20Cº)*:**

Correlation of *k_L_a* and *v_gs_^c^* for oxygen according to [17]:

$$k_{L}\left( O_{2} \right)a=0.32\cdot{{(v}_{gs}^{c})}^{0.7}$$

According to [18], the mass transfer coefficient *k_L_* is dependent on the diameter of the gas bubble *d* and the mean velocity *ω* of the falling or rising bubble and the diffusion coefficient D:

$$k_{L}= {(2}/{\sqrt{\pi})}\cdot\sqrt{D/\left( d/\omega\right)}$$

Assuming that the gas bubble diameter *d* and the mean velocity *ω* are the same for oxygen and the gas species *i*:

$$k_{L}\left( i \right)=k_{L}\left( O_{2} \right)\sqrt{{D\left( i \right)}/{D\left( O_{2} \right)}}$$

Expanding this term by the interfacial area *a*, using the correlation of *k_L_a* and *v_gs_^c^*, leads to the following which allows obtaining the value of *k_L_a* for any gas species:

$$k_{L}a=0.32\cdot{{(v}_{gs}^{c})}^{0.7}\cdot\sqrt{{D\left( i \right)}/{D\left( O_{2} \right)}}$$

**Calculation of electricity generation with BOF gas**

Lower heating value (LHV) of CO: 322 BTU/ft^3^ [19]; CO is assumed to be the only contributor to the heating value of BOF gas.

Conversion to kJ/mol (density of CO: 1.165 kg/m^3^): 288 kJ/mol

1000 m^3^ BOF gas (composition table S1) contains 701 m^3^ CO (29,186 mol) and has a LHV of 8,387,820 (2,330 kWh).

Assuming a combined cycle gas turbine with 56% efficiency for electricity generation [20], that is 1,305 kWh, which equals 104 $ (electricity price of 0.08 kWh/h) or 0.0036 $/mol CO.

References

[1] A. Chauvel and G. Lefebvre, *Petrochemical Processes*: Editions OPHRYS, 1989.

[2] Z. Kapetaki, P. Brandani, S. Brandani, and H. Ahn, “Process simulation of a dual-stage Selexol process for 95% carbon capture efficiency at an integrated gasification combined cycle power plant,” *International Journal of Greenhouse Gas Control*, vol. 39, pp. 17–26, 2015.

[3] *SuperPro Designer®*: Intelligen, Inc, Scotch Plains, NJ, USA.

[4] International Energy Agency, *Renewables 2015.* Available: http://www.iea.org/publications/freepublications/publication/RENTEXT2015_PARTIIExcerpt.pdf.

[5] P. Pei, S. F. Korom, K. Ling, and J. Nasah, “Cost comparison of syngas production from natural gas conversion and underground coal gasification,” *Mitigation and Adaptation Strategies for Global Change*, pp. 1–15, 2014.

[6] J. L. Thompson and W. E. Tyner, “Corn stover for bioenergy production: Cost estimates and farmer supply response,” *Biomass Bioenerg*, vol. 62, pp. 166–173, 2014.

[7] J. L. Thompson and W. E. Tyner, “Corn stover for Bioenergy Production: Cost estimates and Farmer supply response,” https://www.extension.purdue.edu/extmedia/EC/RE-3-W.pdf, 2011.

[8] T. Lin *et al,* “Biomass feedstock preprocessing and long‐distance transportation logistics, *Glob Change Biol Bioenergy*, 2016.

[9] R. M. Swanson, A. Platon, J. A. Satrio, and R. C. Brown, “Techno-economic analysis of biomass-to-liquids production based on gasification,” *Fuel*, vol. 89, pp. S11-S19, 2010.

[10] E. Noor *et al,* “An integrated open framework for thermodynamics of reactions that combines accuracy and coverage,” *Bioinformatics*, vol. 28, no. 15, pp. 2037–2044, 2012.

[11] William M. Haynes, *Handbook of Chemistry and Physics 96th edition.*

[12] E. L. Cussler, *Diffusion, mass transfer in fluid systems, 2nd edition*: New York: Cambridge University Press, 1997.

[13] LMNO Engineering, Research, and Software, Ltd, *Gas Viscosity Calculator.* Available: http://www.lmnoeng.com/Flow/GasViscosity.php.

[14] P.J. Linstrom and W.G. Mallard, Ed, *NIST Chemistry WebBook: NIST Standard Reference Database Number 69*. Gaithersburg MD, 20899.

[15] M. JO, *Perry’s chemical engineers’ handbook*: McGraw Hill, New York, USA.

[16] *Dortmund Data Bank.* Available: www.ddbst.com (2016).

[17] K. Van't Riet and Van der Lans, RGJM, “Mixing in Bioreactor Vessels,” *Comprehensive Biotechnology*, pp. 63–80, 2011.

[18] R. Higbie, “The rate of absorption of a pure gas into still liquid during short periods of exposure,” *Trans. Am. Inst. Chem. Eng*, 1935.

[19] T1 - Section 20 - Consumption A2 - Cleveland, Cutler J and C. Morris, Eds, *Handbook of Energy*. Amsterdam: Elsevier, 2013.

[20] C. Soares, “14 - The Business of Gas Turbines,” in *Gas Turbines*, Burlington: Butterworth-Heinemann, 2008, pp. 557–584.
