# Supplementary material for: Thermodynamics and economic feasibility of acetone production from syngas using the thermophilic production host Moorella thermoacetica
Source: Biotechnol Biofuels. 2017 Jun 12;10:150. doi: 10.1186/s13068-017-0827-8 (PMC5469130; doi:10.1186/s13068-017-0827-8)
Supplement: Supplementary file 2 — Additional file 2. Selection of downstream process scheme. [file 13068_2017_827_MOESM2_ESM.docx]

SUPPLEMENTARY INFORMATION 2

**Selection of downstream process scheme**: The objective is to perform an early-phase estimation of downstream separation costs. This is an active area of research and several options exist to separate the desired chemical from a stream of mixed chemicals. We decided, based on the preliminary runs performed, to explore relative volatility-based separation for this study. Hence, we have narrowed down two distillation schemes illustrated as follows:


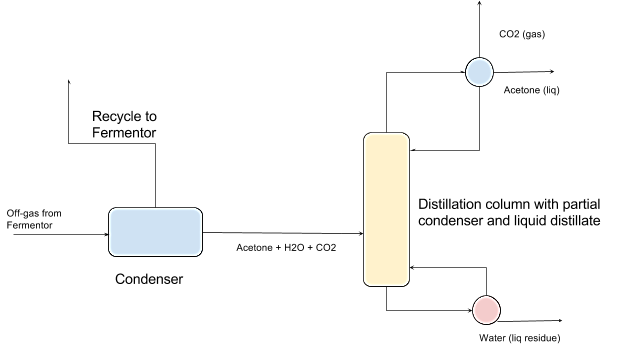


**Figure S2.1.** Single distillation column with partial condenser and a liquid distillate


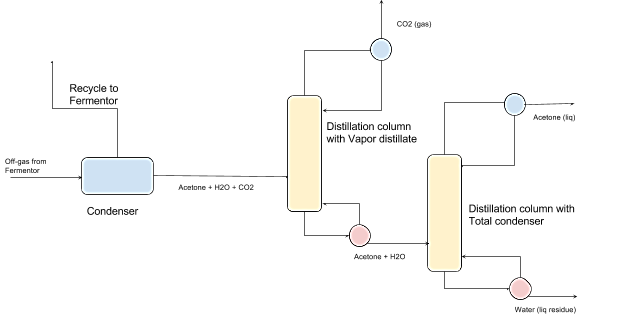


Figure S2.2. Two distillation column configuration.

The condensation operation (common for both cases) of gases aims at removing all major non-soluble gases present in the fermenter off-gas. The off-gas stream comprises CO, CO_2_, H_2_O, N_2_, H_2_ and acetone (the product to be recovered) in composition shown in Table S2.1.

Table S2.1. Composition fermentation off-gas.

| **Components** | **Off-gas composition (kg/hr)** |
| --- | --- |
| Acetone | 2,225 |
| CO_2_ | 11,486 |
| CO | 2,763 |
| H_2_ | 26 |
| N_2_ | 3,045 |
| H_2_O | 2,283 |

Post-condensation, a fraction of the vapor stream is recycled to the fermenter. The condensate (liquid), which mainly comprises acetone, water, and CO_2_ (dissolved), is pumped to the distillation columns. Here, two options are considered to represent a viable separation scheme exploiting the difference in the boiling points of the three compounds. The first scheme consists of one distillation column with a partial condenser with vapor and liquid as distillate. The second configuration consists of a two distillation column set-up which separates CO_2_ in the first column and acetone in the subsequent column as distillation products.

**Simulating the condenser**: in this operation it is desirable to maximize the water/acetone condensate while minimizing the dissolved CO_2_ fraction due to the downstream impact of dissolved or reacted CO_2_ on the subsequent steps. We have set some objectives and constraints to achieve this separation, such as maximizing the acetone in the condensate while keeping the acetone loss minimal. Temperature and pressure were the manipulated variables, while the amount of gas removed and the acetone mole fraction in the condensate and heat duty were tracked. Promising scenarios were found based on multiple runs performed using Aspen Plus® V-8.6.


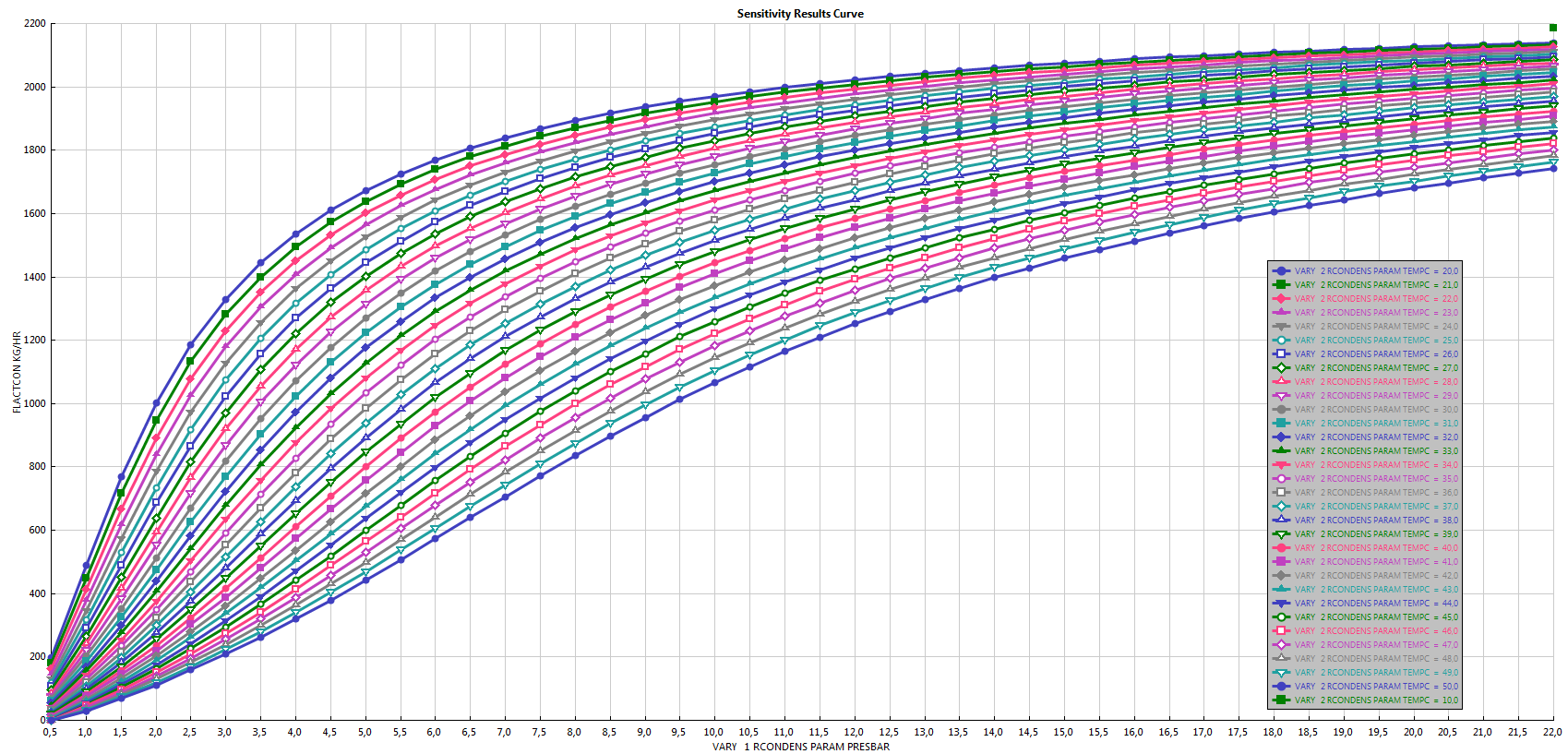


Figure S2.3. Illustration of varying amounts of acetone (y-axis) in the distillate with respect to changing temperature (color code) and pressure (x-axis). At higher pressure and lower temperatures a higher recovery of acetone in the condensate is observed.


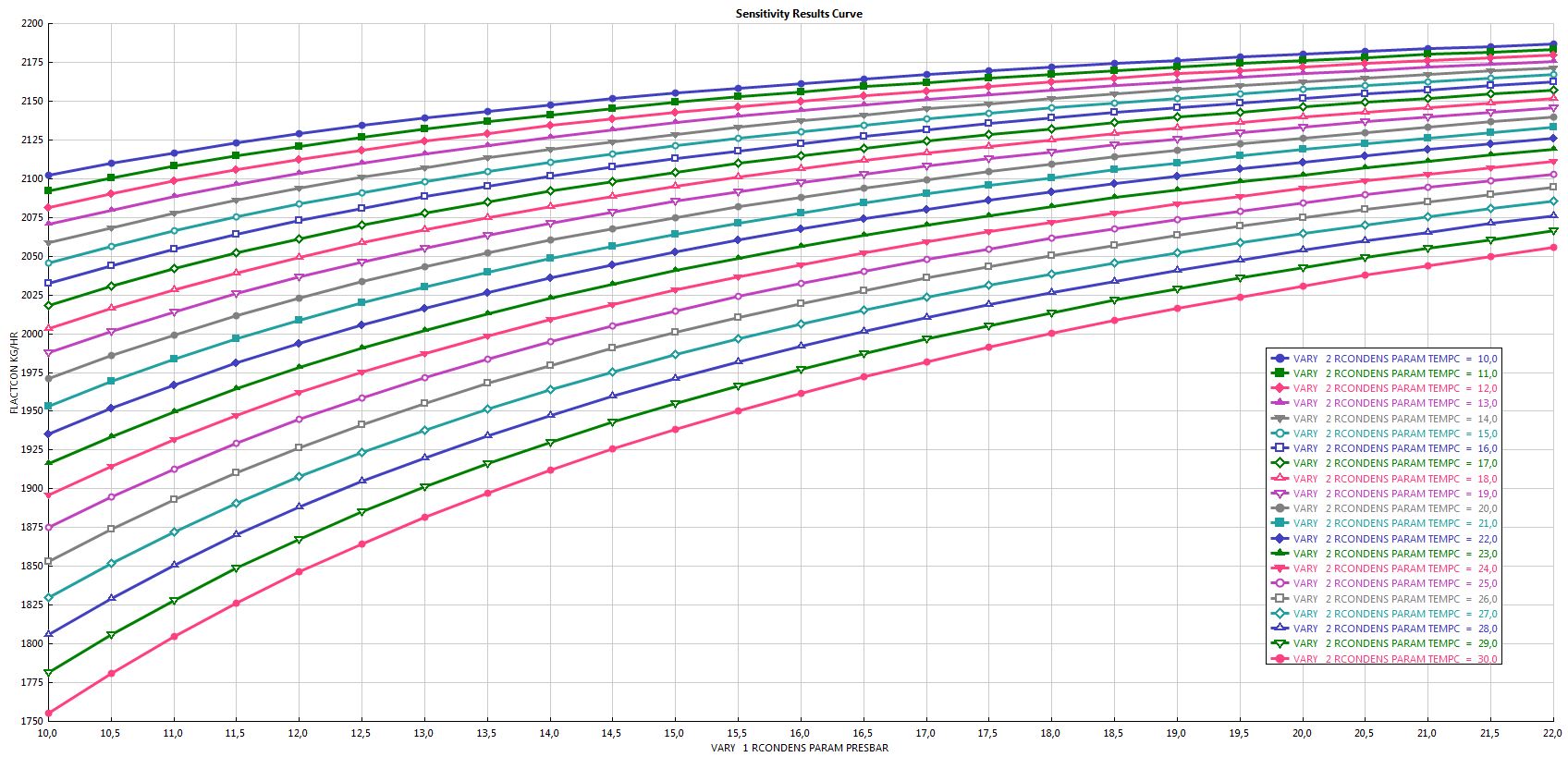


Figure S2.4. The area highlighted in green in figure S2.1. The region above the dotted line shows the conditions at which the recovery of acetone in the condensate is higher than 95% (less than 5% loss).


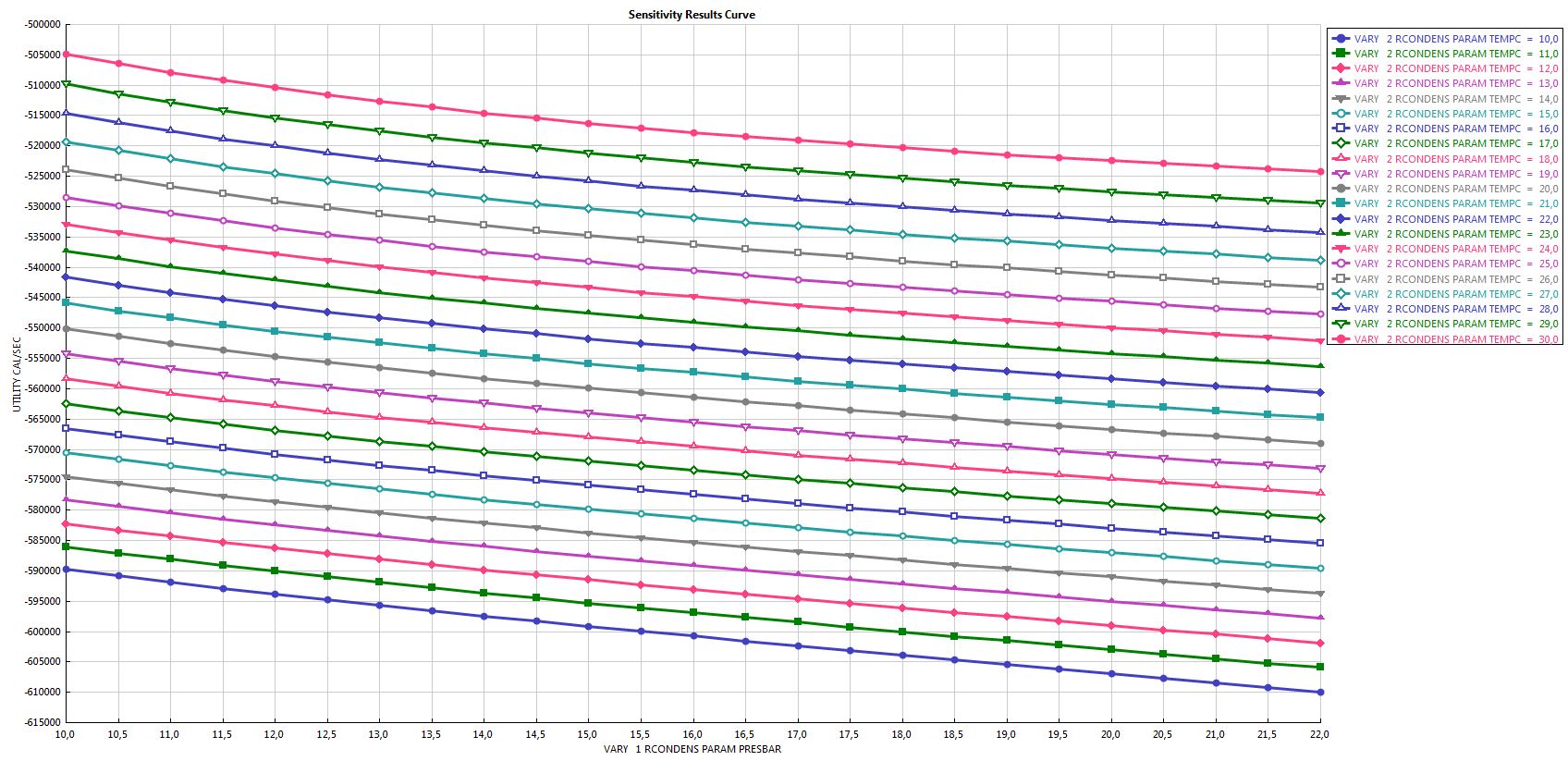


Figure S2.5. Varying duty (y-axis) with respect to conditions in the condenser (x-axis: pressure; color code: temperature).

The parameters, at which the acetone concentration in the condensate is highest, are 10 °C and 22 atm. Subsequent to this step, the product recovery is performed with two distillation columns in series. Hereby a single distillation column is possible, but would lead to a reduction in the purity of the product. The vapor stream from the condenser is at very high pressure (22 bar) and is hence required to pass through a turbine. The generated heat is utilized in the downstream process rather than using a fresh utility. This step is determined based on the conditions prevailing inside the fermenter (60 °C and 364,838 Pa pressure at the bottom of the reactor).

For all the simulations the condenser and distillation operations property methods such as UNIQUAC, NRTL, and WILSON applied. The difference in selected property methods does have some effect on the end results (primarily due to the level of details considered in the calculations performed by each of the mentioned methods). In the tested cases, the variation in acetone concentration was negligible between WILSON and UNIQUAC, whereas the use of NRTL changed the results between 2-3 % when compared to WILSON and UNIQUAC.

**Results from distillation scenarios**: We compared distillation scheme 1 (Figure S2.1) and distillation scheme 2 (Figure S2.2). The best case scenario for one distillation column was able to achieve 1,900 kg/hr in contrast to approximately 2,000 kg/hr for the two distillation column scenario (results shown at the end of this section). Also, there was a trade-off with respect to purity as the single distillation column set up was not able to achieve 99% purity at any given operational range. Hence, for this study a two distillation column set up was used.

**Distillation 1**: The objective was to remove the dissolved CO_2_ using a distillation column. The distillation column was simulated to separate CO_2_ as vapor using a rigorous distillation unit with partial vapor distillate. The bottoms of the column mainly comprise water and acetone.


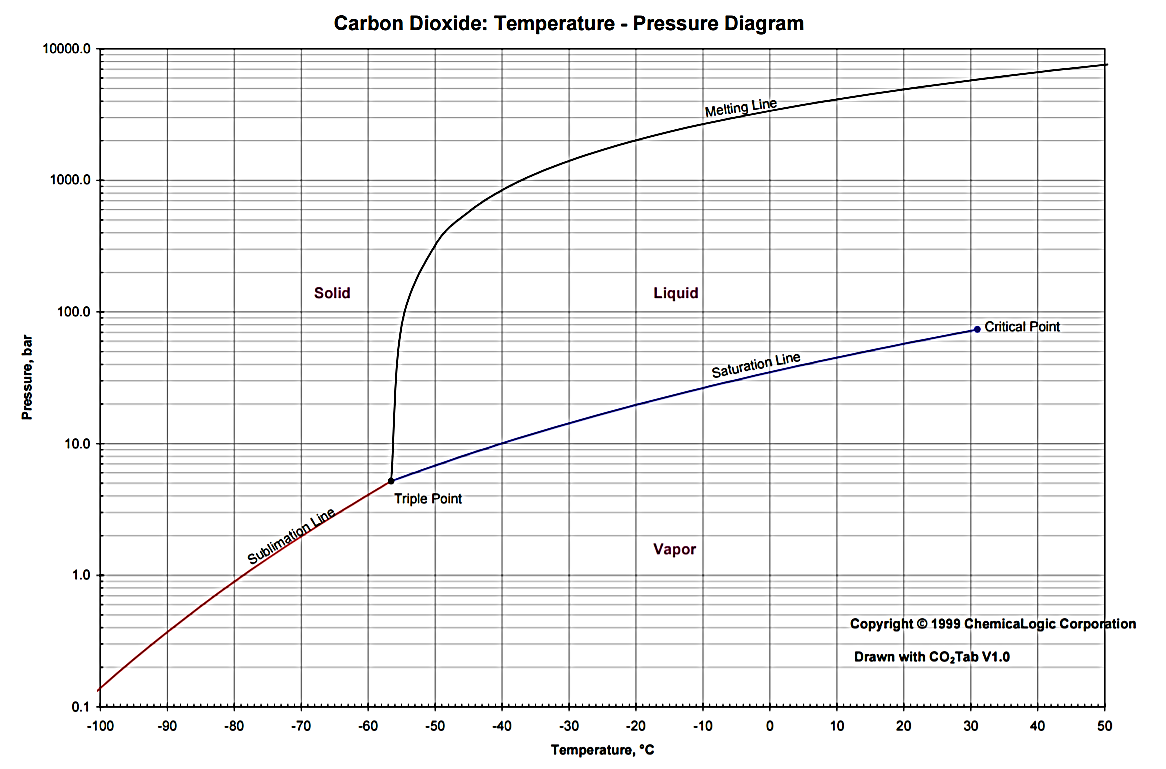


Figure S2.6. In the current runs, CO_2_ is a vapor distillate and is not condensed in the first distillation column. The phase diagram above shows the state of CO_2_ (red dot) in the distillate (vapor), while the green dot represents the state of CO_2_ in the condenser.

**Distillation 2**: The objective was to separate the binary mixture of water and acetone. Acetone is purified and collected as a liquid distillate.

Table S2.2. Column conditions for distillation 1 and distillation 2.

| **Column conditions** | **Distillation 1** | **Distillation 2** |
| --- | --- | --- |
| Property method | UNIQUAC | UNIQUAC |
| Number of stages | 10 | 13 |
| Feed stage | 4 | 11 |
| Reflux ratio | 0.05 | 1.82 |
| Condenser pressure (atm) | 1 | 0.5 |
| Condenser temperature (°C) | -18 | 37 |
| Condenser utility | Refrigerant | Cooling water |
| Reboiler pressure | 2 | 1.5 |
| Reboiler temperature (°C) | 89 | 109 |
| Reboiler utility | Steam (low pressure) | Steam (low pressure) |

Table S2.3. The product stream attributes for the simulated cases for the 2-column configuration.

| **Distillate stream** | **Mass Flow** in kg/hr | **Mass fraction** |
| --- | --- | --- |
| Acetone | 2,058 | 99% |
| CO_2_ | 0 | 0% |
| CO | 0 | 0% |
| H_2_ | 0 | 0% |
| N_2_ | 0 | 0% |
| H_2_O | 19.69 | 1% |
| Carbonic | 0 | 0% |

Table S2.4. Utility summary.

| **Details** | **Operating Utility Contributions** | **Utility consumed** in kW | **Cost** in $ |
| --- | --- | --- | --- |
| Condensation | Off-gas compression | 2,705 | 216 |
|  | Off-gas cooler | 5,141 | 93 |
|  | Condenser column | 411 | 7 |
| Distillation 1 | Condenser | 27 | 2 |
|  | Reboiler | 888 | 18 |
| Distillation 2 | Condenser | 906 | 3 |
|  | Reboiler | 666 | 14 |
|  | **Total Utility** | **10,744** | **353** |

Table S2.5. Cost contribution for downstream processes.

| **Downstream Section** | **Cost** in $ |
| --- | --- |
| Condensation | 316.34 |
| Distillation 1 | 20.06 |
| Distillation 2 | 16.93 |
| **Total** | **353.34** |
